# Low-dose interleukin-2 alleviates dextran sodium sulfate-induced colitis in mice by recovering intestinal integrity and inhibiting AKT-dependent pathways

**DOI:** 10.7150/thno.41534

**Published:** 2020-04-06

**Authors:** Hana Lee, Ye Seul Son, Mi-Ok Lee, Jea-Woon Ryu, Kunhyang Park, Ohman Kwon, Kwang Bo Jung, Kwangho Kim, Tae Young Ryu, Aruem Baek, Janghwan Kim, Cho-Rok Jung, Choong-Min Ryu, Young-Jun Park, Tae-Su Han, Dae-Soo Kim, Hyun-Soo Cho, Mi-Young Son

**Affiliations:** 1 Korea Research Institute of Bioscience and Biotechnology (KRIBB), 125 Gwahangno, Yuseong-gu, Daejeon 34141, Republic of Korea;; 2 KRIBB School of Bioscience, Korea University of Science and Technology (UST), 217 Gajeong-ro, Yuseong-gu, Daejeon 34113, Republic of Korea;; 3 Department of Biology, Chungnam National University, Daejeon, 34134, South Korea

**Keywords:** ulcerative colitis, interleukin-2, dextran sulfate sodium, PI3K-AKT pathway, RNA-sequencing

## Abstract

Several phase 1/2 clinical trials showed that low-dose interleukin-2 (IL-2) treatment is a safe and effective strategy for the treatment of chronic graft-versus-host disease, hepatitis C virus-induced vasculitis, and type 1 diabetes. Ulcerative colitis (UC) is a chronic inflammatory condition of the colon that lacks satisfactory treatment. In this study, we aimed to determine the effects of low-dose IL-2 as a therapeutic for UC on dextran sulfate sodium (DSS)-induced colitis.

**Methods**: Mice with DSS-induced colitis were intraperitoneally injected with low-dose IL-2. Survival, body weight, disease activity index, colon length, histopathological score, myeloperoxidase activity and inflammatory cytokine levels as well as intestinal barrier integrity were examined. Differential gene expression after low-dose IL-2 treatment was analyzed by RNA-sequencing.

**Results**: Low-dose IL-2 significantly improved the symptoms of DSS-induced colitis in mice and attenuated pro-inflammatory cytokine production and immune cell infiltration. The most effective dose range of IL-2 was 16K-32K IU/day. Importantly, low-dose IL-2 was effective in ameliorating the disruption of epithelial barrier integrity in DSS-induced colitis tissues by restoring tight junction proteins and mucin production and suppressing apoptosis. The colon tissue of DSS-induced mice exposed to low-dose IL-2 mimic gene expression patterns in the colons of control mice. Furthermore, we identified the crucial role of the PI3K-AKT pathway in exerting the therapeutic effect of low-dose IL-2.

**Conclusions**: The results of our study suggest that low-dose IL-2 has therapeutic effects on DSS-induced colitis and potential clinical value in treating UC.

## Introduction

Ulcerative colitis (UC), one of the major types of inflammatory bowel disease (IBD), is a chronic recurrent inflammatory disease characterized by damage to the mucosal epithelium and disruption of intestinal homeostasis 1. Although various studies suggest that genetic background, environmental factors and immunological dysfunction contribute to the development of UC, the pathogenesis of UC remains poorly understood 2, 3. Patients with UC tend to develop various degrees of inflammation in extra-intestinal tissues, including the liver, lungs, skin, eyes, joints, and vascular system 4. Moreover, a number of patients with UC are at high risk of developing colorectal cancer 5.

Nuclear factor kappa-light-chain-enhancer of activated B cells (NF-κB) activation and increased levels of pro-inflammatory cytokines, particularly tumor necrosis factor-alpha (TNF-α), interleukin-6 (IL-6), and IL-1β, have been detected in the intestinal epithelium of UC patients 6, 7. Genetic polymorphisms and serum levels of IL-17 contribute to the development and progression of UC 8. Secreted pro-inflammatory cytokines including TNF-α and IL-6, as well as reactive oxygen species generated by recruited immune cells as well as reactive oxygen species generated by recruited immune cells such as neutrophils, subsequently lead to intestinal epithelial apoptosis, which promotes mucosal inflammation and results in loss of barrier integrity by reducing the level of tight junction (TJ) proteins in patients with UC and IBD 9, 10. Thus, repair of the mucosal barrier and suppression of inflammation are effective strategies for the treatment of UC 11. Conventional drugs for UC, including corticosteroids, aminosalicylates, and antibiotics 12, as well as anti-TNF-α therapies, are widely used in clinical settings 12, 13. However, these drugs have limitations including substantial economic burden and undesirable side-effects; consequently, an effective and safe treatment for UC patients is urgently needed.

IL-2 is known as a T cell growth factor, controlling the proliferation and differentiation of T cells 14. Based on this function, IL-2 therapy has been used to stimulate an immune response to increase T cell numbers* in vivo*, particularly in treating cancers and human immunodeficiency virus-infected patients 15-17. Treatment with relatively high doses of IL-2 have beneficial effects *in vivo*; however, they often lead to side effects such as liver and kidney dysfunction and vascular leakage syndrome that limit the clinical use of such high doses 18. In addition to the administration of IL-2, the monoclonal antibody basiliximab, which binds with high affinity to the α chain of the IL-2 receptor (CD25), has been shown to prevent rejection after organ transplantation 19. IL-2 is also known to play key roles in the development and maintenance of CD4^+^ regulatory T cells (Tregs) that function to negatively regulate immune-mediated inflammation and stimulate tissue repair processes 20-22.

Furthermore, when administered at low doses, IL-2 is reported to confer protective effects against chronic autoimmune inflammation 23. A recent study showed that low-dose IL-2 selectively expanded Tregs and attenuated symptoms in mice with trinitrobenzene sulfonic acid/dinitrobenzene sulfonic acid (TNBS/DNBS)-induced colitis 24. Phase 1/2 clinical studies showed the safety of applying low-dose IL-2 in the treatment of patients with chronic graft-versus-host disease (GVHD) 25, hepatitis C virus (HCV)-induced vasculitis 26, and type 1 diabetes (T1D) 27.

Nonetheless, the therapeutic mechanism of action of IL-2 on inflammation associated with UC remains unknown. Studies are necessary to investigate the direct effects of low-dose IL-2 on the intestinal epithelium in colitis in order to determine the appropriate dose of IL-2. Herein, we evaluated the therapeutic efficacy of low-dose IL-2 in dextran sulfate sodium (DSS)-induced experimental colitis in mice. Furthermore, possible mechanisms by which low-dose IL-2 exerted its action, as well as the detailed outcomes of such treatments were investigated in the colons of mice with DSS-induced colitis, primarily the affected sites in UC.

## Materials and Methods

### Animals

Male C57BL/6J mice at 7-8 weeks of age (20-22 g) were purchased from the Jackson Laboratory (Bar Harbor, ME, USA). All experiments on animals were approved by the Institutional Animal Care and Use Committee (IACUC) of the Korea Research Institute of Bioscience and Biotechnology (Approval No: KRIBB-AEC-19216). The mice were group-housed with standard animal maintenance at a constant temperature (20-22 °C) under 12 h day/night cycles.

### DSS-induced colitis and IL-2 treatment

The DSS-induced colitis model involved the administration of drinking water with 3% (w/v) DSS (36-50 kDa; MP Biomedicals, Hampton, NH, USA) for 7 days. The DSS+PBS and DSS+IL-2 group received fresh water for 4 days after receiving 3% DSS for 7 days and the control mice received fresh water for 11 days. The mice were segregated into Control, DSS+IL-2 (IL-2 dose: 1.6 × 10^3^, 8.0 × 10^3^, 1.6 × 10^4^, 3.2 × 10^4^, and 8.0 × 10^4^ IU/day, respectively), and DSS+PBS groups. The DSS+IL-2 groups was injected intraperitoneally with recombinant murine IL-2 (PeproTech, Rocky Hill, NJ, USA) daily for 7 days (day 4 to day 10) of treatment and the DSS+PBS group received an intraperitoneal injection of PBS daily from day 4 to day 10 of treatment. The DSS+IL-2 and DSS+PBS groups were injected with 200 μl for 7 days. Body weights were monitored daily for 11 days, and mice with a body weight loss of more than 25% were considered to have reached the experimental endpoint 28. On day 11, the mice were sacrificed, their abdomens were opened and the entire colon (from the caecum to the anus) was removed. To assess IL-2 toxicity, the control group was injected intraperitoneally with recombinant murine IL-2 for 7 days. On day 11, mice were sacrificed and the entire colon was removed. To observe the reverting effect of IL-2, the control and DSS PBS groups were prepared as above, and the DSS+IL-2 group was observed for an additional 7 days after intraperitoneal injection of IL-2 into DSS-induced colitis mice for 7 days. On day 18, the mice were sacrificed to remove the entire colon from the abdomen. To confirm the long-term effect of IL-2, DSS-induced colitis mice were intraperitoneally injected with IL-2 for 14 days and 21 days and followed up. On day 18 and day 25 respectively, mice were sacrificed and the entire colon, small intestine, spleen, kidney, lung and liver were harvested and examined.

### Evaluation of clinical scoring

Assessment of the disease activity index (DAI) was determined by the scoring system described in Table S1 29. Weight loss, stool consistency, and severe bleeding parameters were assessed.

### Histopathologic assessment

After the mice were sacrificed, colon length as well as the macroscopic properties of the colon were measured. For histopathological analysis, colon tissues were fixed in 10% formalin and incubated in sucrose solution for cryopreservation. The tissues were embedded in Tissue-Tek^®^ O.C.T compound (Sakura^®^ Finetek, Tokyo, Japan) and cut into 5-μm sections. The sections were stained with hematoxylin & eosin (H&E). Histopathological scores were determined according to previously described criteria 29. Briefly, the histopathological scores of the colonic lesions were assessed by the degree of inflammation, infiltration of neutrophils and lymphocytes, crypt damage, sub-mucosal edema, and loss of goblet cells (Table S2). The pathological scores were obtained by adding all of the scores from the parameters described above.

### Myeloperoxidase (MPO) activity assay

To evaluate neutrophil infiltration into the colon, MPO activity was measured in the supernatant from colon tissues using an MPO assay kit (Abcam, Cambridge, UK) according to the manufacturer's instructions. Tissues were incubated in 1 mL Dulbecco's modified Eagle's medium (DMEM; Gibco^®^/Thermo Fisher Scientific, Carlsbad, CA, USA) containing 0.2% fetal bovine serum (FBS, Invitrogen/Thermo Fisher Scientific, Carlsbad, CA, USA) and 1% penicillin-streptomycin (P/S; Gibco) in 24-well plates (Corning, NY, USA) at 37 ℃ for 24 h. The results are expressed in the following units (U): MPO/mg of protein.

### Quantitative reverse transcription PCR (RT-qPCR)

Total RNA was extracted using TRIzol™ reagent (Thermo Fisher Scientific, Waltham, MA, USA) and cDNA synthesis was performed using the Super-Script™ IV First-strand Synthesis System (Invitrogen/Thermo Fisher Scientific) as described previously 30. RT-qPCR was performed using Brilliant III Ultra-Fast SYBR^®^ Green QPCR Master Mix and the signal was evaluated on an AriaMx Real-Time PCR System (Agilent Technologies, Inc., Santa Clara, CA, USA). Gene expression was normalized against beta-actin levels. The primers used in this study are listed in Table S3.

### Reverse transcription PCR (RT-PCR)

PCR was performed under the followed conditions: 3 min at 94 °C then 32-40 cycles of RT-PCR (94 °C for 30 s, 55-60 °C for 40 s, 72 °C for 40 s), and 72 °C for 5 min for elongation. PCR products were analyzed using electrophoresis on 2% agarose gel using SYBR Safe DNA gel stain (Invitrogen) and a Gel-Doc (Bio-Rad, Hercules, California, USA).

### Enzyme-linked immunosorbent assay (ELISA)

The colon tissues were cut into smaller pieces (< 0.5 cm) and washed with DMEM (Invitrogen) supplemented with 0.2% FBS (Invitrogen) and 1% P/S (Invitrogen). The tissues were incubated in 1 mL DMEM containing 0.2% FBS and 1% P/S in 24-well plates (Corning) at 37 ℃. After 24 h, the supernatant and tissues were harvested. To analyze the changes in inflammatory cytokine production, the supernatant samples were used to measure the levels of TNF-α, IL-6, IL-1β, and IL-17 (R&D Systems, Minneapolis, MN, USA). The measured values were normalized to the total protein concentration.

### Immunohistochemistry and immunofluorescence

Immunohistochemical staining was performed on formalin-fixed colon tissues as described previously 31, 32. Tissue sections were permeated with 0.1% Triton X-100 and then blocked for 1 h with 4% bovine serum albumin. After blocking, sections were incubated with primary antibodies (Table S4) overnight at 4 ℃. For chromogenic detection, the sections were then incubated for 1 h at room temperature (RT) with the appropriate secondary antibody. Horseradish peroxidase activity was detected by 3,3′diaminobenzidine (DAB; IHC WORLD, Woodstock, MD, USA), which acts as a chromogen, for 30 seconds. Cell nuclei were stained with hematoxylin. Chromogenic intensity was measured using FIJI/ImageJ 33. For immunofluorescence staining, after incubation with primary antibodies, tissue sections were incubated with secondary antibodies for 1 h at RT then counterstained with 4′,6-diamidino-2-phenylindole (DAPI). Between each step, tissues were washed three times with PBS for 5 min. The fluorescence was visualized using an EVOS microscope (FL Auto 2, Thermo Fisher Scientific, Inc.).

### Alcian blue-periodic acid-Schiff (AB-PAS) and mucicarmine staining

To detect mucin-secreting goblet cells, AB-PAS and mucicarmine staining were performed. Alcian blue (AB, Abcam), periodic acid-Schiff (PAS; MilliporeSigma, Burlington, MA, USA), and mucicarmine (Abcam) stain kits were applied to sectioned tissue slides following the manufacturers' recommended protocols.

### RNA-sequencing (RNA-seq) and analysis

Total RNA integrity was assessed on an Agilent 2100 Bioanalyzer system (Agilent Technologies) using an RNA 6000 Nano Kit (Agilent). RNA purification and library construction for RNA-seq were performed according to the TruSeq RNA Sample Preparation Kit V2 (Illumina, San Diego, CA USA). Sample sequencing was performed on Illumina HiSeq 2500 machines (Illumina) using the standard Illumina RNA-seq protocol with a read length of 2 × 100 base pairs (bp). The quality of the paired-end reads was evaluated with FastQC v 0.11.4 and filtered to remove low quality reads (Phred quality score > 20) and adapter sequences with Cutadapt v.1.15 and Sickle v.1.33. If the trimmed read length was less than 50 bp, it was excluded. Filtered reads were then mapped to the Mus musculus reference genome (mm10) with HISAT2 v.2.1.0 using default parameters. To obtain quantification scores for all mouse genes and transcripts, Fragments Per Kilobase of transcripts per Million mapped reads (FPKM) values were calculated using Cufflinks version 2.2.1, which corrects for transcript length and total number of mapped reads from each library to compensate for different read depths for different samples. Differentially expressed genes (DEGs) were analyzed using CuffDiff in the Cufflinks package (*P* < 0.05 and fold-change > 1.5) as described previously 34. Fold-change was recalculated using the FPKM+1 value of each gene to avoid infinite values. All of the Gene Ontology and Kyoto Encyclopedia of Genes and Genomes (KEGG) pathway enrichment analyses were performed with Database for Annotation, Visualization and Integrated Discovery (DAVID) v.6.8. Correlation plots, hierarchical clustering, heat maps, and principal component analysis (PCA) were analyzed using R script (http://www.r-project.org).

### Western blot analysis

The colon tissues were homogenized and lysed with RIPA buffer supplemented with 1× PMSF (Thermo Fisher Scientific), 1 mM protease inhibitor (Sigma-Aldrich, St. Louis, MO, USA) and 1× phosphatase inhibitor (Sigma-Aldrich) as described previously 35. Protein samples (30 µg each) were separated on precast 4-20% gradient gels (Bio-Rad Laboratories, Hercules, CA, USA). Proteins were transferred to activated polyvinylidene difluoride membranes (Bio-Rad Laboratories) and blocked with 5% skim milk at RT for 1 hr. The primary antibodies were incubated at 4 ℃ overnight. After washing, the membranes were incubated with HRP-conjugated secondary antibodies at RT for 2 h. The bands were measured on an LAS-3000 imager (Fujifilm, Tokyo, Japan). The list of primary antibodies used is provided in Table S4.

### Isolation of mouse intestinal immune cells and FACS analysis

Mouse lamina propria Isolation experiments were performed after approval by the Institutional Animal Care and Use Committee (IACUC) of KRIBB (approval No: KRIBB-AEC-19216). Ten weeks-old C57BL/6 J mice (Dae Han Bio Link Co., Ltd., Eumseong-gun, Chungcheongbuk-do, Korea) were used. For analysis of mouse colon lamina propria immune cells, mouse intestinal neutrophils and T cells were isolated using previously reported methods 36 from the control, DSS+PBS, DSS+IL-2 (1.6 × 10^4^, 3.2 × 10^4^ IU/day) group mice. The colon lamina propria immune cells were stained with surface antibodies at 4 °C for 1 h (Table S4). For intracellular staining, the cells were washed, fixed, and permeabilized with Fixation/permeabilization solution (BD Biosciences, San Diego, CA, USA). After washing with staining buffer, the cells were incubated the intracellular antibody at 4 °C for 1 h. The cells were analyzed with Accuri C6 (BD Biosciences) and FlowJo V10 software (FLOWJO, Ashland, OR, USA.

### Mouse intestinal neutrophil sorting and viability assay

The isolated intestinal neutrophils were sorted using the MojoSort™ Mouse Neutrophil Isolation Kit (Biolegend, San Diego, CA, U.S.) following the manufacturer's protocol. The neutrophils were incubated with Iscove's Modification of DMEM (Corning) contained 10% FBS (Gibco), 1× P/S (Gibco), 1× L-glutamine (Gibco), 1× MEM NEAA (Gibco), and 2 mM cAMP (Enzo life sciences, Farmingdale, NY, USA). The media was supplemented 160, 1.6 × 10^4^, or 16 × 10^4^ IU /ml IL-2. After incubation, the cells were stained with Trypan Blue solution (Gibco), and the live and dead cells were counted.

### Isolation and culture of mouse colonoids (mCOs) derived from DSS-induced colitis mice

Pieces of colon (~0.5 mm) from the control and DSS-induced colitis mice were incubated for 30 min at room temperature on an orbital rocker in crypt chelating buffer. Colonic crypts were isolated and cultured, as previously reported 37. The isolated crypts were cultured in the IntestiCult™ Organoid Growth Medium (STEMCELL technologies, Vancouver, BC, Canada) with murine recombinant Wnt3a (R&D Systems). The medium was changed every 2 days, and mIOs were passaged every week. To assess the effects of IL-2, mCOs were treated with recombinant murine IL-2 (10-1600 IU/ml, Peprotech) for 7 days at passage 0. The morphology of mCOs was visualized using microscopy (EVOS FL Auto 2, Thermo Fisher Scientific, Inc.) until passage 1. On day 14, mCOs were processed for RT-qPCR analysis. The growth of mCOs was evaluated by measuring the surface area.

### Statistical analysis

All experiments were repeated at least three times and results are expressed as means ± standard error (SEM). One-way ANOVA and a two-tailed t-test were used to analyze the significance of the data statistically.

## Results

### Low-dose IL-2 ameliorates the clinical signs of DSS-induced colitis

**Figure 1:**
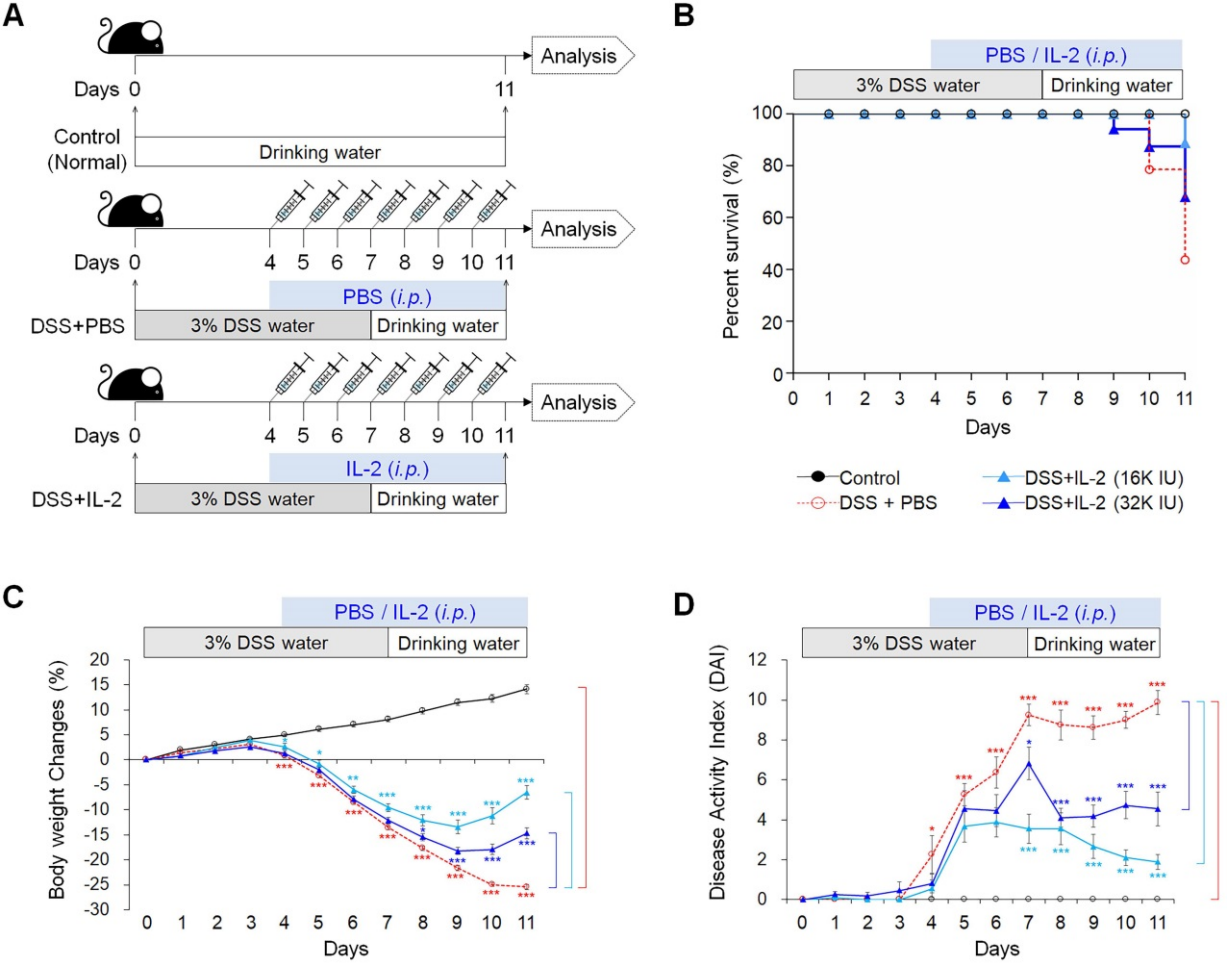
** Low-dose IL-2 ameliorates clinical signs in mice with DSS-induced colitis.** The groups of mice used in the study were Control (n = 25), DSS+PBS (n = 40), DSS+IL-2 (16K IU, n = 30), and DSS+ IL-2 (32K IU, n = 28). **(A)** Schematic diagram of the experimental design. **(B)** Kaplan-Meier survival analysis. **(C)** Percentage body weight change. **(D)** Disease activity index (DAI), scored from body weight loss, stool, and bleeding. Data are presented as mean values of replicates ± SEM. ***p < 0.001, **p < 0.01, and *p < 0.05 using one-way ANOVA with a *post hoc* analysis and t-test.

To examine the effect of low concentrations of IL-2 in an experimental colitis model, we induced acute colitis in mice by adding 3% DSS to their drinking water for 7 days. Mice were injected daily with various concentrations of IL-2 or PBS intraperitoneally starting from four days after the first DSS administration, when significant weight loss was observed until the end of the experiment on day 11 (Figure 1A). DSS exposure resulted in a survival rate of 43.7%, whereas the survival rate of mice with DSS-induced colitis was significantly improved in the low-dose IL-2 groups (the survival rates for 16K IU/day and 32K IU/day recombinant IL-2-groups were 88.9% and 68.0%, respectively) (Figure 1B). The survival rate of mice treated with lower and higher doses of IL-2 (55.5% for 1.6K IU/day, 58.9% for 8K IU/day, and 68.8% for 80K IU /day) was lower than that of the mice in the low-dose IL-2 groups (Figure S1A). In the DSS group, a reduction in body weight was recorded after 4 days of DSS exposure, with the weight loss remaining significantly higher than that recorded in the control group until day 11 (31.1 ± 0.9%; *P* < 0.001; Figure 1C and Figure S1B). The low-dose IL-2 groups (16K IU/day and 32K IU/day) significantly improved the body weight in mice with colitis compared to the DSS+PBS group (*P* < 0.001; Figure 1B). Another common feature of the DSS‑induced model of colitis is an increase in the DAI (28). Only low-dose IL-2 groups significantly reduced cumulative DAI with normal stool consistency and no rectal bleeding compared to the DSS+PBS group (Figure 1D and Figure S1C).

### Effect of low-dose IL-2 on colon length and histopathology of mice with DSS-induced colitis

**Figure 2:**
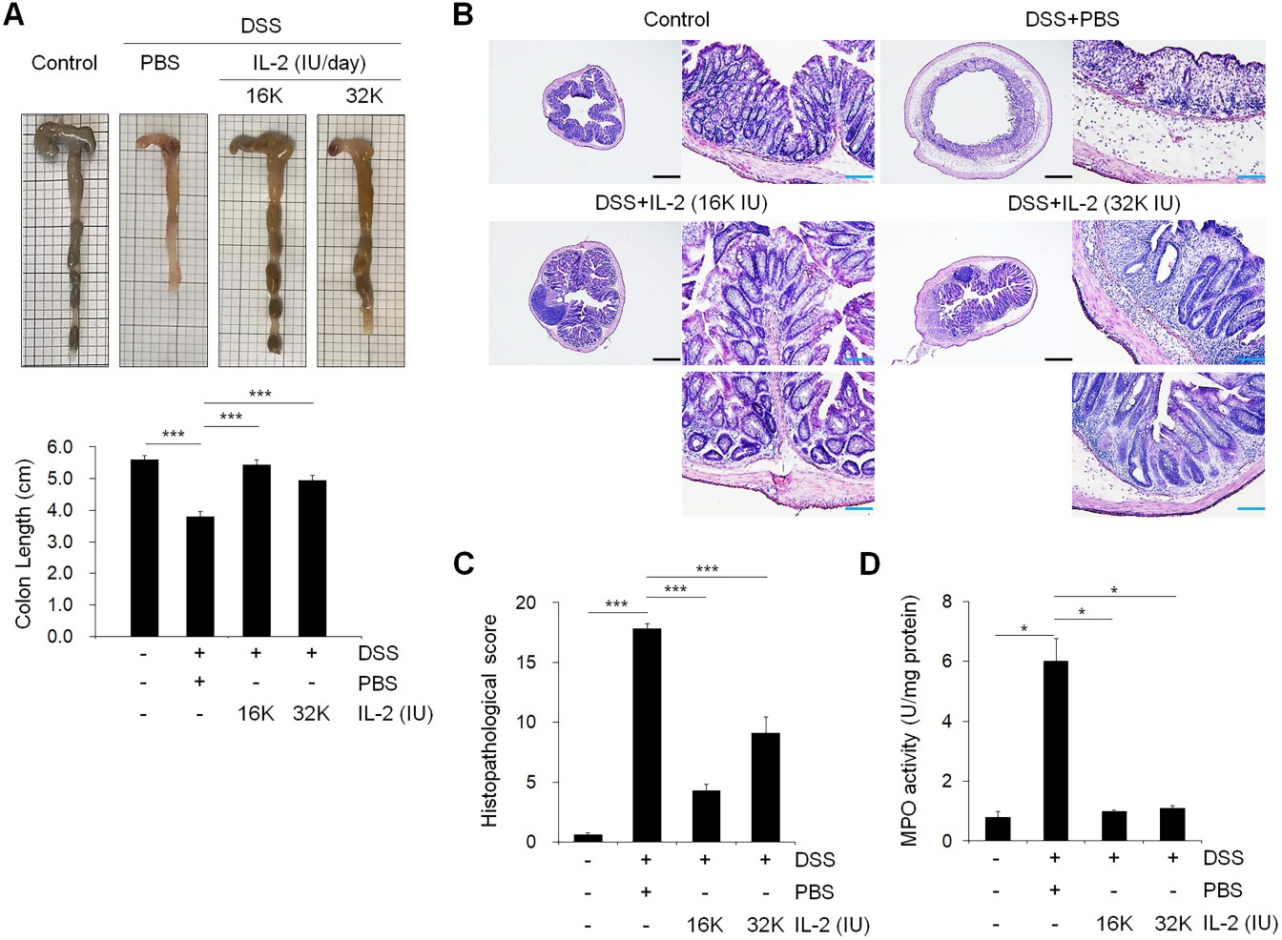
** Effect of low-dose IL-2 on the histopathology of DSS-induced colitis. (A)** Representative photographs of the colon (upper) and changes in colon length (bottom) (n = 10 for each group). **(B)** Histopathological analysis in colon tissue by H&E staining. Scale bar, black 500 µm and blue 100 µm. **(C)** Histopathological changes scored for colon tissue (n = 10 for each group). **(D)** Myeloperoxidase (MPO) activity of colon tissue. Data are presented as mean values of replicates ± SEM. ***p < 0.001, **p < 0.01, and *p < 0.05 according to t-test.

To examine the effect of low-dose IL-2 on inflammation-induced shortening in colon length, we evaluated the morphology of colonic inflammation in a DSS‑induced colitis mouse model 38. The colon length in DSS-induced mice was significantly shorter compared to that in the control mice (*P* < 0.001; Figure 2A). The mice treated with DSS and low-dose IL-2 (16K IU/day and 32K IU/day) exhibited a significant increase in colon length when compared to the DSS+PBS group (*P* < 0.001; Figure 2A). The colon length in both the lower and higher IL-2 dose groups was higher than those in the DSS+PBS group (8K IU/day,* P* < 0.01 and 80K IU/day,* P* < 0.05; Figure S2). IL-2 treatment at low-doses (16K IU/day and 32K IU/day) significantly improved the histological alterations caused by DSS, as shown in representative images in Figure 2B and the histopathological score in Figure 2C. Low-dose IL-2, i.e., 16K IU/day and 32K IU/day, produced more protective effects against inflammation-induced mucosal injury than the lower and higher doses of IL-2 (1.6K IU/day, 8K IU/day, and 80K IU /day) (Figure S3A). Treatment with various concentrations of IL-2 by itself had no significant effect on the histopathology in control mice over the entire 11 day monitoring period (Figure S4). Furthermore, to quantify neutrophil infiltration into the colon tissues, the activity of MPO, a marker of neutrophil granulocytes was significantly decreased in low-dose IL-2 groups compared to the DSS+PBS group (*P* < 0.001; Figure 2D and S3B). The reduction of neutrophils in the colon tissue after low-dose IL-2 treatment may be due to the reduction of infiltration, and not by selective killing, as evidenced by data showing no direct effect of low-dose IL-2 on apoptosis and survival of neutrophils and the downregulated expression of genes involved in chemokine signaling pathway and CXC chemokine genes, which play an essential role in the recruitment of neutrophils 39, 40, after treatment with low-dose IL-2 (Figure S5). We also showed that the symptoms of colitis were not reverted even after IL-2 activity was reduced (Figure S6). These results imply that the effects of low-dose IL-2 are stable in mice after a 7-day treatment period, followed by 7 days of no treatment. Additionally, after the long-term administration of low-dose IL-2 (14 days and 21 days), the symptoms of colitis induced in mice by DSS were efficiently relieved without detrimental effects in other organs (Figure S7 and S8). Taken together, these results suggested that low-dose IL-2 reduced the signs of DSS-induced colitis in mice.

### Low-dose IL-2 suppressed pro-inflammatory responses in DSS-induced colitis in mice

**Figure 3:**
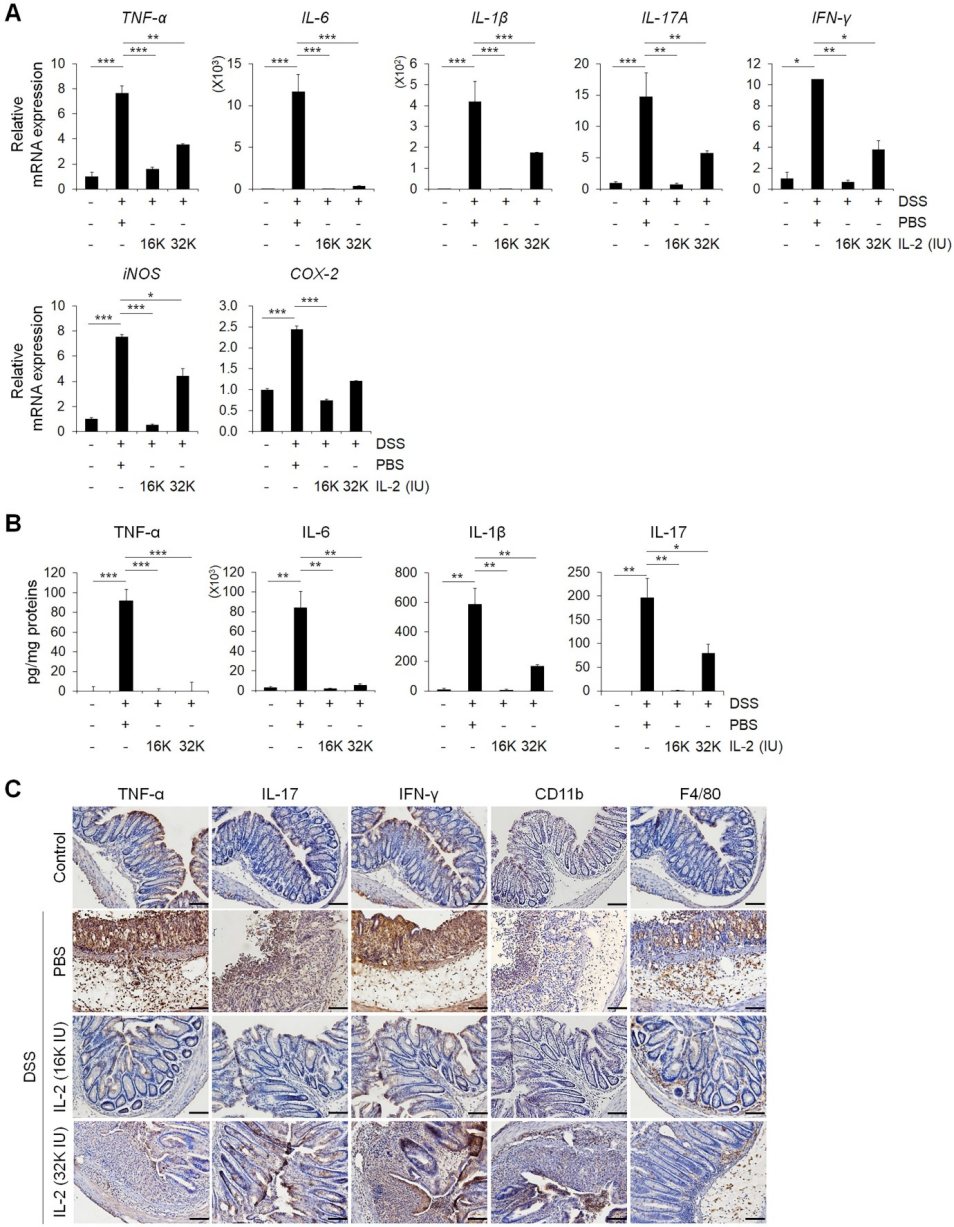
** Low-dose IL-2 suppressed pro-inflammatory markers in the colons of mice with DSS-induced colitis. (A)** The relative expression levels of inflammatory cytokines (*TNF-α, IL-6, IL-1β, IL-17A, and IFN-γ*) and inflammatory enzymes (*COX-2* and *iNOS*) in colon tissue were assessed by qPCR. (n ≥ 5 for each group). **(B)** ELISA quantification of TNF-α, IL-6, IL-1β, and IL-17 concentration in the supernatant of colon tissue cultured for 1 day (n ≥ 5 for each group). **(C)** Immunohistochemistry of pro-inflammatory cytokines TNF-α, IL-17, and IFN-γ, myeloid cells (CD11b), and macrophages (F4/80). Red-brown color indicates the protein expression of TNF-α, IL-17, IFN-γ, CD11b, and F4/80. The tissue was counterstained with hematoxylin. Scale bar, 100 µm. Data are presented as mean values of replicates ± SEM. ***p < 0.001, **p < 0.01, and *p < 0.05 according to t-test.

To evaluate the effects of low-dose IL-2 on pro-inflammatory responses in the colons of mice with DSS-induced colitis, we analyzed the levels of inflammatory cytokines and enzymes whose overexpression can cause oxidative stress, thereby inducing apoptosis and aggravating the symptoms of DSS-induced colitis 1, 41, 42. The gene expression levels of the major inflammatory cytokines including *TNF-α*, *IL-6*, *IL-1β*, *IL-17A*, and interferon-gamma (*IFN-γ*) increased in the DSS+PBS group were significantly downregulated by low-dose IL-2 (16K IU/day and 32K IU/day) (*P* < 0.05; Figure 3A and Figure S9). In addition, gene expression levels of the inflammatory enzymes in colon inducible nitric oxide synthase (*iNOS*) and cyclooxygenase-2 (*COX-2*) were decreased to the control level within 7 days after low-dose IL-2 treatment (Figure 3A). The secretory levels of TNF-α, IL-6, IL-1β, and IL-17 were significantly increased after DSS treatment, while low-dose IL-2 treatment decreased the elevated levels of these pro-inflammatory cytokines in the supernatant of colon tissues cultured for one day (Figure 3B). Thus, low-dose IL-2 exerts an anti-colitis effect by inhibiting the gene expression and secretion of pro-inflammatory factors. There is also a marked difference in cytokine levels between 16K IU and 32K IU of IL-2. This result was consistent with that 16K IU (1μg/day) was more effective in alleviating symptoms of colitis than 32K IU (2μg/day), as evidenced by the improvement in the clinical signs of 16K IU (1μg/day) treated group.

Next, we used immunohistochemistry to evaluate protein expression levels of the major pro-inflammatory cytokine, myeloid cell (CD11b positive), and macrophage (F4/80 positive) markers in colon tissues (Figure 3C) 43. In DSS-induced colitis, levels of the major pro-inflammatory cytokines including TNF-α, IL-17, and IFN-γ, were higher than those in the control and low-dose IL-2 groups (16K IU/day and 32K IU/day) (Figure 3C). DSS treatment increased the number of myeloid cells (CD11b positive) and macrophages (F4/80 positive) that infiltrated into the mucosal and epithelial layers of the damaged colon compared to that of control mice. The infiltration of CD11b and F4/80 positive cells in the colon was dramatically suppressed in low-dose IL-2-treated mice (Figure 3C). These results imply that treatment with low-dose IL-2 may play a role in DSS-induced colonic inflammation by attenuating the production of pro-inflammatory cytokines and infiltration of immune cells.

### Low-dose IL-2 restored intestinal barrier function in experimental colitis models *in vivo*

**Figure 4:**
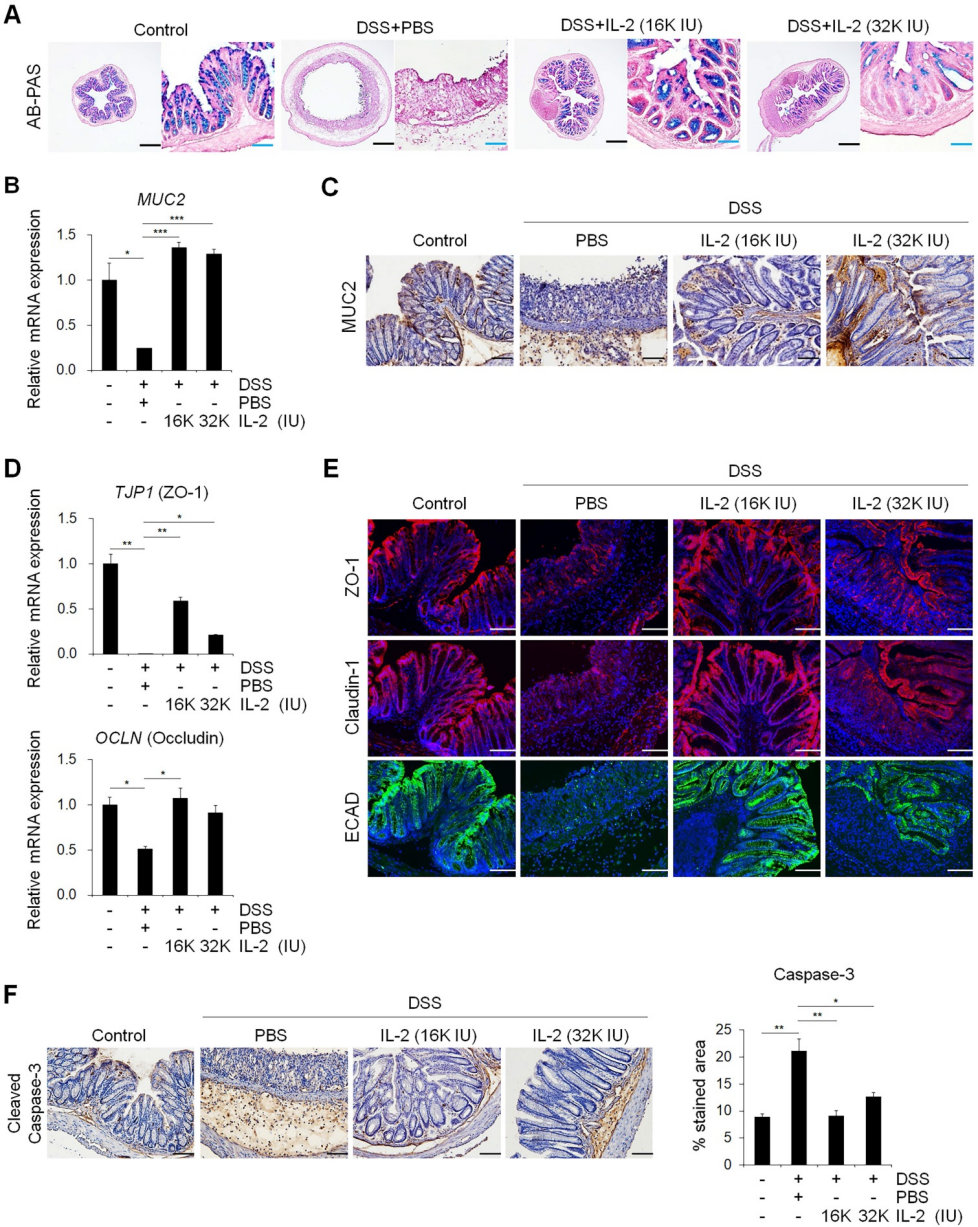
** Low-dose IL-2 restored the functions of goblet cells and defects of the intestinal barrier. (A)** To detect mucin, colon tissue was stained with AB-PAS staining. Scale bar, black 500 µm and blue 100 µm. **(B)** The mRNA expression level of *MUC2* in colon tissue was determined by qPCR; n = 5 per group. **(C)** Mucin (MUC2) protein expression in colon tissue was detected by immunohistochemistry. Scale bar, 100 μm. **(D)** The relative expression levels of tight junction markers (*ZO-1* and *Occludin*) in colon tissue were assessed by qPCR; n = 5 per group. **(E)** Immunofluorescent staining of Control, DSS+PBS, and DSS+IL-2 with tight junction (ZO-1, claudin-1) and cell-cell adhesion markers (ECAD). Nuclei were stained with DAPI (blue). Scale bar, 100 µm. **(F)** Immunohistochemistry of intestinal epithelial apoptosis marker (cleaved caspase-3, left). Scale bar 100 µm. Quantification of DAB-stained area with Fuji ImageJ (n = 3 for each group). Data are presented as mean values of replicates ± SEM. ***p < 0.001, **p < 0.01, and *p < 0.05 according to t-test.

Mucins produced by goblet cells provide a frontline of intestinal defense against enteric pathogens; changes in mucin levels influence epithelial barrier function, which in turn leads to chronic inflammation and an elevated cancer risk 44, 45. Next, we investigated whether IL-2 can modulate the function of goblet cells in colitis. AB-PAS and mucicarmine staining revealed that treatment with low-dose IL-2 increased the quantity and function of goblet cells, revealing higher mucus levels (Figure 4A and Figure S3C and S3D). Treatment with low-dose IL-2 significantly increased the expression levels of both MUC2 mRNA and protein in colon tissue compared to the DSS+PBS group (Figure 4B and 4C). In a mouse model of DSS-induced acute colitis, disruption of epithelial barrier function is a representative symptom associated with impairment of the TJ proteins. Therefore, we investigated the effect of low-dose IL-2 on the expression of TJ molecules by qPCR and immunofluorescence analyses. In the DSS-induced colitis group, there were substantial reductions in *zonula occludens-1 (ZO-1)* and *occludin* mRNA levels; however, these recovered to the control levels in the low-dose IL-2 groups (16K IU/day and 32K IU/day) (Figure 4D). Next, we examined the suppressive effect of low-dose IL-2 on the reduction in TJ protein and E-cadherin (ECAD) levels, which is also important in regulating epithelial integrity 46. Immunofluorescence analysis showed that DSS treatment markedly reduced the expression of ZO-1, claudin-1, and E-cadherin in the intestinal epithelial layer, whereas treatment with low-dose IL-2 led to higher expression levels of ZO-1, claudin-1, and E-cadherin (Figure 4E). It has also been reported that DSS-induced colonic epithelial cell apoptosis contributes to the disruption of intestinal integrity 47. We found that treatment with low-dose IL-2 decreased DSS-induced intestinal epithelial apoptosis by immunohistochemistry to active caspase-3 (Figure 4F). These results imply that low-dose IL-2 positively modulates DSS-induced damage by blocking the loss of intestinal barrier-related molecules in inflamed and damaged colon tissue. Furthermore, IL-2 treatment at low-doses has little effect on the expansion of CD4^+^/FOXP3^+^ Tregs in the colon tissue of DSS-induced mice (Figure S10). Rather, it is likely that IL-2 can directly affect the colonic epithelium at specific concentrations, as evidenced by the increased growth of mCOs and the expression of intestinal cell markers and barrier markers, through its receptor expressed on colonic epithelial cells using mCO culture isolated from the DSS-induced colitis mouse model (Figure S11).

### Treatment with low-dose IL-2 induced a transcriptome change in the colons of mice with DSS-induced colitis

**Figure 5:**
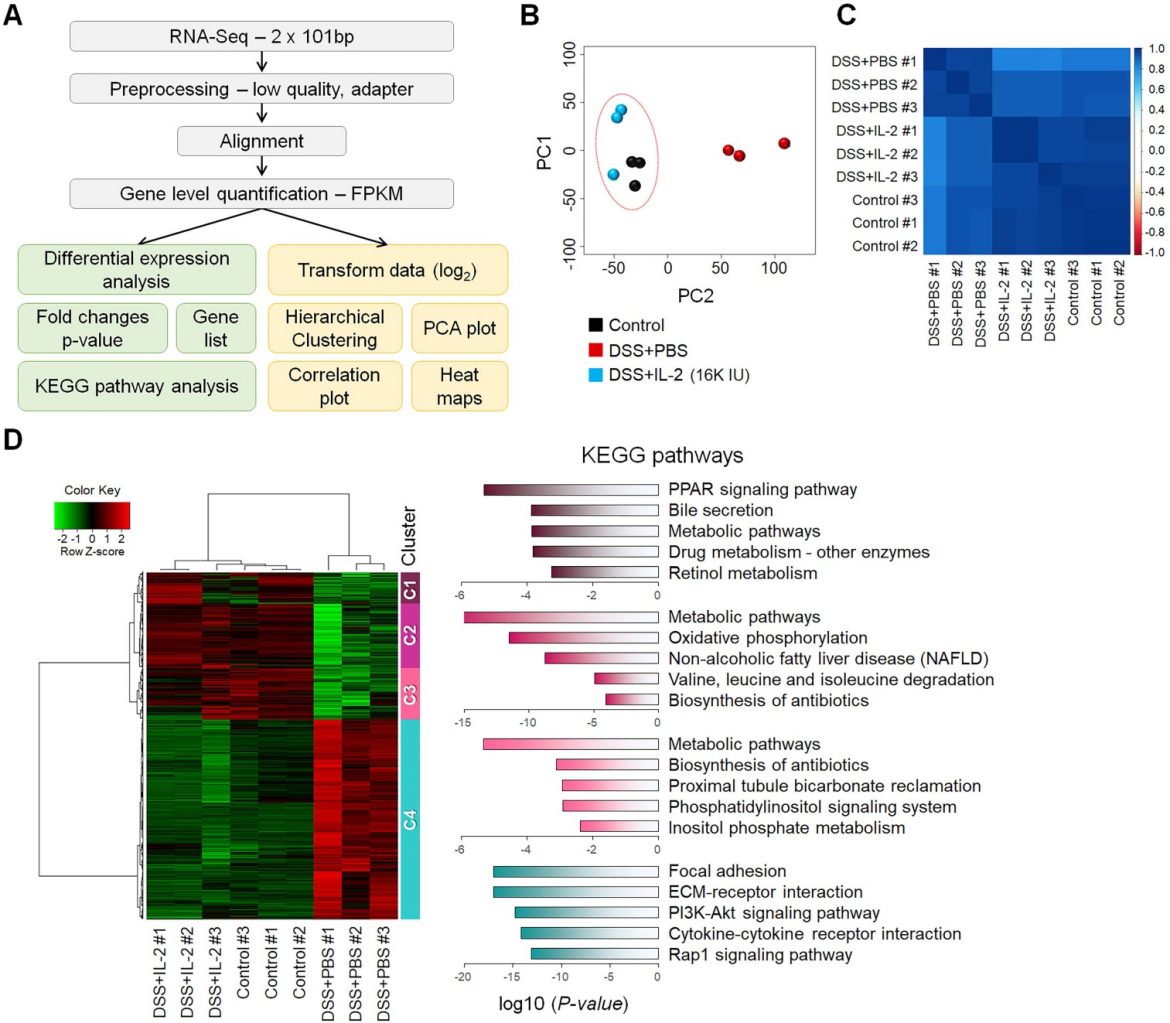
** Transcriptomic analysis of mice with DSS-induced colitis exposed to low-dose IL-2. (A)** The workflow for differential expression analysis based on the RNA-seq data. **(B)** Principal component analysis (PCA) was performed with the top 500 highest expressed genes for each sample. Control and DSS+IL-2 samples are closely clustered. (n = 3 for each group). **(C)** A correlation plot was used to cluster samples and generate a heatmap. Dark blue indicates the highest level of similarity between samples. Control and DSS+IL-2 samples showed a highly positive correlation. **(D)** Hierarchical clustering of 3,393 overlapping genes was performed using the hclust function and the ward.D2 method (left). Red indicates upregulated genes and green indicates downregulated genes. The representative pathway of genes belonging to each cluster is indicated by a bar graph, according to the *p*-value.

To better understand the treatment effects of IL-2 in mice with DSS-induced colitis, we performed RNA-seq analysis of the mouse colon samples obtained from the control, DSS+PBS, and DSS+IL-2 (16K IU/day) (Figure 5A). All experiments were performed in triplicates. An average of 96% of reads aligned in at least one region of the reference genome. We calculated the abundance levels for each gene (measured as FPKM). In the RNA-Seq results for nine samples, 15,808 genes were identified with a sum of all samples greater than 1 FPKM value. We compared the identified DEGs among the control, DSS+PBS, and DSS+IL-2 (16K IU/day) groups. Our analysis revealed 4,249 significant DEGs (17.7%) in the DSS+PBS group compared to the controls, all of which are related to UC. We identified 4,578 significant DEGs in the DSS+IL-2 group compared to the DSS+PBS group, all of which are experimentally identified IL-2 treatment-related genes. PCA based on global transcriptomic data revealed that the control (in black) and DSS+PBS group (in red) were clearly separated and the transcriptome of the DSS+IL-2 group was distinct from that of the DSS+PBS group and tended to be closer to the transcriptome of the control group (Figure 5B). The correlation plot shows that the transcriptomes of the DSS+IL-2 group resembled more closely to the profile of the control group than that of the DSS+PBS group (Figure 5C). We further identified 3,393 genes that overlapped between colitis-related and IL-2 treatment -related genes and the resulting heatmap was divided into five clusters (Figure 5D, left panel). Moreover, 1,443 genes among those downregulated and 1,950 genes among those upregulated were found to have altered gene expression patterns skewed toward the gene expression patterns in the control group following IL-2 treatment (IL-2-responsive genes), as shown by the heatmap displaying partial restoration of the gene expression pattern after IL-2 treatment (Figure 5D, left panel). Pathway enrichment analysis was performed for each cluster to identify significant pathways perturbed by DSS-induced colitis and restored by low-dose IL-2. KEGG pathway analysis showed that the genes that were most significantly differentially expressed between the DSS+PBS and DSS+IL-2 groups were those related to PPAR signaling (Cluster 1), metabolic pathways (Clusters 2 and 3), and focal adhesion and ECM-receptor interaction (Cluster 4) (Figure 5D, right panel). Together, these results show that low-dose IL-2 treatment alleviated the defective phenotypes of DSS-induced colitis to some degree at both the cellular and molecular levels *in vivo*.

### Low-dose IL-2 alleviates experimental colitis by modulating PI3K/AKT and NF-κB pathways

**Figure 6:**
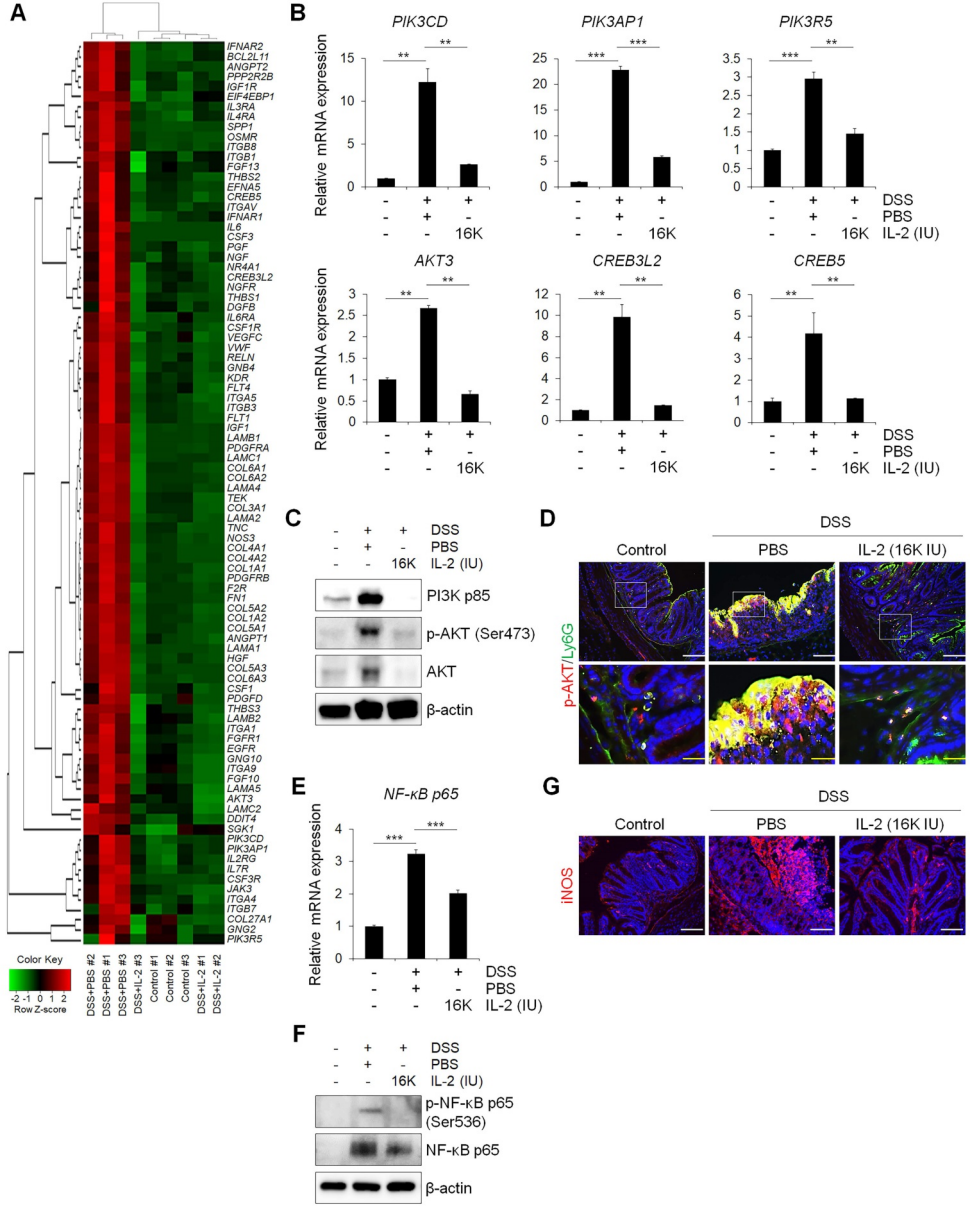
** Low-dose IL-2 relieves experimental colitis by regulating the PI3K/AKT and NF-κB pathways. (A)** Heatmap of genes known to be involved in the PI3K/AKT pathway from RNA-seq data. Red indicates upregulated genes, while green indicates downregulated genes. **(B)** qPCR analysis of genes involved in the PI3K/AKT pathway in colon tissue (n = 3 for each group). **(C)** Representative western blot analysis of PI3K p85, phospho-AKT (Ser473), and AKT levels in colon tissue. **(D)** Immunofluorescent staining of Control, DSS+PBS, and DSS+IL-2 (16K IU/day) with p-AKT and Ly6G. Scale bar, white 50 µm and yellow 25 µm. **(E)** qPCR analysis of the expression of *NF-κB p65* (n = 3 for each group). **(F)** Representative western blot analysis of phospho-NF-κB p65 (Ser536) and NF-κB p65. **(G)** Immunofluorescent staining with iNOS. Nuclei were stained with DAPI (blue). Scale bar, 100 µm. Data are presented as mean values of replicates ± SEM. ***p < 0.001 and **p < 0.01 according to t-test.

We sought to determine whether the therapeutic effect of low-dose IL-2 in DSS-induced colitis is associated with phosphoinositide 3-kinase (PI3K)-protein kinase B (AKT) downregulation, given that the PI3K-AKT pathway was significantly enriched in Cluster 4—the largest cluster—which was upregulated in the DSS+PBS group and downregulated by treatment with low-dose IL-2 (16K IU/day). Expression changes for genes involved in the PI3K-AKT pathway predominantly shifted toward the control group based on RNA-seq analysis (Figure 6A). The RT-qPCR results revealed that the expression pattern of PI3K-AKT pathway-associated genes was similar to that observed in the RNA-seq analysis (Figure 6B). The expression levels of PI3K, phospho-AKT (Ser473), and AKT were increased in DSS-induced colon tissues as determined by western blot analysis, consistent with previous studies 48. However, low-dose IL-2 treatment attenuated these high expression levels in the colonic tissue of DSS-treated mice; furthermore, the infiltrating immune cells, such as the neutrophils, were the main contributors toward the high expression levels of PI3K, phospho-AKT, and AKT (Figure 6C and 6D). The activation of PI3K/AKT is also important to stimulate the NF-κB signaling pathway, which is upregulated in IBD patients, promoting the expression of various pro-inflammatory genes, which especially exacerbates mucosal inflammation 49, 50. Gene expression of *NF-κBp65* was upregulated in DSS-induced colitis and suppressed by low-dose IL-2 treatment (16K IU/day; Figure 6E). Consistently, there was a significant increase in NF-κBp65 and phospho-NF-κBp65 (Ser536) in the colon tissue of DSS-induced mice which was prevented when these mice were treated with IL-2 (16K IU/day; Figure 6F). The expression of iNOS, which is regulated by NF-κB, increased in DSS-induced colitis and was reduced by treatment with low-dose IL-2 back to the basal levels observed in the control group (Figure 6G). Furthermore, the gene expression levels of PI3K-AKT were increased after treatment with certain concentrations of IL-2 in the mCOs isolated from DSS-induced colitis mice (Figure S12), consistent with a previous report that IL-2 signaling induces PI3K-AKT-mediated survival 51. Therefore, we inferred that the reduced expression level of PI3K, p-AKT, and AKT in colonic tissue from DSS-treated mice upon low-dose IL-2 treatment might be due to reduced immune cell infiltration to the colonic tissues and not solely dependent on reduced colonic epithelial cell apoptosis. These data suggest that low-dose IL-2 exerts an inhibitory effect on the PI3K/AKT and NF-κB pathways *in vivo* in ameliorating the disease phenotype of DSS-induced colitis. The gene levels associated with the common gamma chain-jak-stat pathways, which are known to be induced by IL-2, were also examined in colon tissue from DSS-induced colitis mice. Gene levels of IL-2Rγ, Jak3, Stat5a/b and Stat3 were significantly increased (Figure S13A) and the results were consistent with RNA-seq (Figure S13B). These results imply that other signaling pathways may also be involved in the effects of low-dose IL-2 on the intestinal epithelium in colitis.

## Discussion

In this study, we demonstrated that low physiological concentrations of IL-2 ameliorated clinical disease activity in mice with DSS-induced colitis. Specifically, low concentrations of IL-2 reduced pro-inflammatory cytokine production in the colons of mice with DSS-induced colitis and improved intestinal barrier integrity. In addition, we showed that low-dose IL-2 has a therapeutic effect by regulating the expression of PI3K/AKT and NF-κB in mice with DSS-induced colitis.

The first clinical use of high-dose IL-2 (Proleukin^®^) was approved to treat renal cell carcinoma in 1992 and metastatic melanoma in 1998 52. However, only a limited number of patients (< 10%) respond successfully to such treatment and the adverse effects of high-dose IL-2 limit its therapeutic use 17. Recently, it was reported that low-dose IL-2 can selectively stimulate and expand Tregs, which are beneficial in facilitating recovery for patients with autoimmune diseases such as T1D, rheumatoid arthritis, and systemic lupus erythematosus, as well as other diseases such as HCV-induced vasculitis and chronic GVHD 23, 26, 53, 54. In a very recent report, a group of researchers demonstrated that low-dose IL-2 expanded Tregs in the blood and spleen* in vivo* and attenuated DNBS/TNBS-induced colitis in mice; they recently conducted a phase 1b/2a clinical trial of low-dose IL-2 therapy, investigating its safety and therapeutic efficacy in UC patients with moderate-to-severe disease activity 24. It is well known that low-dose IL-2 treatment improves the survival and expansion of peripheral blood Tregs 26, 55-57; however, it was reported that there was no difference in Tregs at the site of inflammation in the colon before and after treatment with low dose IL-2 24, consistent with our data. Therefore, although the function of Tregs could be improved by treating with low-dose IL-2, an evaluation of the functional effects of low-dose IL-2 on the intestinal epithelium itself and an investigation of the mode of action of low-dose IL-2 on intracellular signaling pathways *in vivo* are necessary for the development of an effective UC therapy.

Although the precise pathophysiological mechanisms of UC remain unclear, it has been suggested that the loss of intestinal barrier integrity by a dysregulated immune response can lead to the development of UC 58. Consequently, the maintenance of intestinal integrity is a crucial component of an effective therapeutic strategy for UC. Loss of intestinal integrity causes severe inflammation; furthermore, inflamed sites contain a greater number of apoptotic cells, resulting in delayed intestinal regeneration 59. We demonstrated that treatment with low-dose IL-2 blocked the intestinal tissue damage otherwise caused by DSS-induced colitis in mice (Figure 4). It is more evident that low-dose IL-2 can directly affect the colonic epithelium through its receptor, as demonstrated by the use of mCO, a culture system of colonic epithelial cells, which was consistent with previous studies demonstrating that IL-2 can directly modulate epithelial functions, such as cell proliferation and secretion of intestinal epithelial cells only at specific concentrations 60-62. These results signify that low-dose IL-2 decreased inflammation in the damaged intestinal epithelium, which is likely to be caused by the low-dose IL-2-induced protection of the intestinal junction. On the other hand, high IL-2 dose is reported to induce toxicity and apoptosis in several cell types, such as intestinal epithelial cells 61 and pancreatic beta cells 63, thereby disrupting the homeostasis of intestinal and pancreatic tissues. Therefore, it seems that IL-2 plays a role in the control of cell proliferation and death in a concentration-dependent manner.

Epithelial TJs play an essential role in the maintenance of the intestinal barrier, preventing the penetration of inflammatory molecules and also regulating the permeability of nutrients, ions, and water. The loss of epithelial barrier integrity in acute colitis is associated with impairment of the TJ proteins (consisting of a variety of proteins including ZO proteins, claudins, occludin, and junctional adhesion molecules) 64 and increased epithelial apoptosis 65. Consistent with IBD patient data 59, the disruption of ZO-1 and E-cadherin was observed in DSS-induced colitis (Figure 4D and 4E). We also found that the barrier-protective effects of low-dose IL-2 are associated with TJs and the decreased colonic epithelial cell apoptosis (Figure 4F). Muc2-deficient mice are known to develop a spontaneous colitis phenotype with mucus layer depletion, one of the characteristic features of patients with UC 66, and high levels of pro-inflammatory cytokines 67. Mice with DSS-induced colitis displayed reduced levels of MUC-2 expression and mucin secretion, which is restored by low-dose IL-2 treatment (Figure 4A-C). Thus, these protective roles of low-dose IL-2 may mediate its treatment effects on DSS-induced colitis by inhibiting the loss of junctional molecules and reducing intestinal epithelial apoptosis in damaged intestinal tissue.

Based on our RNA-seq analysis results, mice with DSS-induced colitis treated with low-dose IL-2 exhibited the differential expression of a specific set of genes in the colon, in particular, those associated with the PI3K-AKT signaling pathway—compared to mice treated with PBS (Figure 5). It has been reported that the PI3K/AKT and NF-κB pathways contribute to cell proliferation, survival, angiogenesis, and apoptosis 68. Consistent with our data (Figure 6D), a previous study also showed that p-AKT is strongly expressed by the infiltrating immune cells 48, implying that PI3K-AKT activation mainly occurs in infiltrated immune cells and triggers the inflammatory responses through immune cell activation. PI3K-dependent AKT phosphorylation is a key event in the progression of UC via the activation of inflammatory signaling 48. AKT was reported to be more phosphorylated in colonic biopsies of IBD patients and experimental *in vivo* models of colitis; moreover, the severity of colitis was reduced when treated with PI3K inhibitors, wortmannin and AS605240 48, 69. We revealed the presence of enhanced phosphorylated and total forms of AKT in the colon of mice treated with DSS, which was decreased to control levels by low-dose IL-2 treatment (16K IU/day; Figure 6C and 6D). Activation of the NF-κB-modulated infiltration of immune cells in the colon and rectal mucosa is believed to contribute to the development of UC 70; furthermore, the expression and phosphorylation of NF-κB p65 increased in a DSS-induced colitis model 71. There have been many previous reports of crosstalk between PI3K/AKT and NF-κB pathways involved in the protection and decreased disease activity in experimental model of colitis 49, 72, 73. It was also reported that the PI3K/AKT pathway is an upstream regulator of NF-κB 74-77. Considering the extensive regulatory functions of PI3K/AKT with downstream molecules, activation of not only the PI3K/AKT pathway and but also the NF-κB pathway can enhance the production of pro-inflammatory cytokines 38, 78. We found that low-dose IL-2 (16K IU/day) reduced the expression and phosphorylation level of NF-κB p65 protein, thus inhibiting NF-κB activity (Figure 6F). The subsequent induction and expression of various inflammatory cytokines (TNF-α, IL-6, IL1β, IL-17, and IFN-γ) and enzymes (iNOS and COX-2) that are associated with the initiation and development of colitis 49, 79 were also prevented by low-dose IL-2 (Figure 3). However, the results from the* in vitro* mCOs culture model in the absence of immune cells suggest that PI3K-AKT signaling may be involved in cell survival in epithelial cells at specific concentrations through its receptor expressed on epithelial cells. Further studies are needed to clarify the mechanisms of the relationship between intestinal epithelial cells and immune cells involved in intestinal regeneration in DSS-induced colitis. Additionally, other signaling pathways might also contribute to the effects of low-dose IL-2 on the intestinal epithelium in colitis, such as the common gamma chain-Jak-Stat pathways, which are known to be important in the enhancement of proliferation and intestinal barrier regeneration at specific concentrations of IL-2 acting through the IL-2R on the intestinal epithelium 61, 62, 80.

In conclusion, low-dose IL-2 effectively improved DSS-induced colitis symptoms in mice, resulting in the overall attenuation of histological changes, expression and secretion of pro-inflammatory cytokines, and immune cell infiltration. Our experiments demonstrated that low-dose IL-2 ameliorates DSS-induced intestinal tissue damage by restoring intestinal barrier integrity and exerting an inhibitory effect on the PI3K/AKT and NF-κB pathways* in vivo*, thereby protecting the intestinal epithelium against DSS-induced colitis. Low-dose IL-2 is expected to offer a promising therapy for diseases associated with intestinal barrier damage such as UC and Crohn's disease.

## Supplementary Material

Supplementary figures and tables.

## Abbreviations

AB: Alcian blue
AB-PAS: Alcian blue-periodic acid-Schiff
AKT: protein kinase B
COX-2: cyclooxygenase-2
DAB: 3,3′diaminobenzidine
DAI: disease activity index
DAPI: 4′,6-diamidino-2-phenylindole
DEGs: Differentially expressed genes
DMEM: Dulbecco's modified Eagle's medium
bp: base pairs
DSS: dextran sulfate sodium
ECAD: E-cadherin
ELISA: Enzyme-linked immunosorbent assay
FPKM: Fragments Per Kilobase of transcripts per Million mapped reads
GVHD: graft-versus-host disease
H&E: hematoxylin & eosin
HCV: hepatitis C virus
IACUC: Institutional Animal Care and Use Committee
IBD: inflammatory bowel disease
IFN-γ: interferon-gamma
IL: interleukin
iNOS: inducible nitric oxide synthase
IU: international units
KEGG: Kyoto Encyclopedia of Genes and Genomes
mCO: mouse colonoid
MPO: Myeloperoxidase
NF-kB: Nuclear factor kappa-light-chain-enhancer of activated B cells
P/S: penicillin-streptomycin
PAS: periodic acid-Schiff
PCA: principal component analysis
PI3K: Phosphoinositide 3-kinase
RNA-seq: RNA-sequencing
RT: room temperature
RT-qPCR: Quantitative reverse transcription PCR
SEM: means ± standard error
T1D: type 1 diabetes
TJ: tight junction
TNBS/DNBS: trinitrobenzene sulfonic acid/dinitrobenzene sulfonic acid
TNF-α: tumor necrosis factor-alpha
Tregs: CD4^+^ regulatory T cells
U: units
UC: Ulcerative colitis
ZO: zonula occludens
