# Supplementary material for: Low-dose interleukin-2 alleviates dextran sodium sulfate-induced colitis in mice by recovering intestinal integrity and inhibiting AKT-dependent pathways
Source: Theranostics. 2020 Apr 6;10(11):5048–63. doi: 10.7150/thno.41534 (PMC7163458; doi:10.7150/thno.41534)
Supplement: Supplementary file 1 — Supplementary figures and tables. [file thnov10p5048s1.pdf]

Supplementary figures and tables

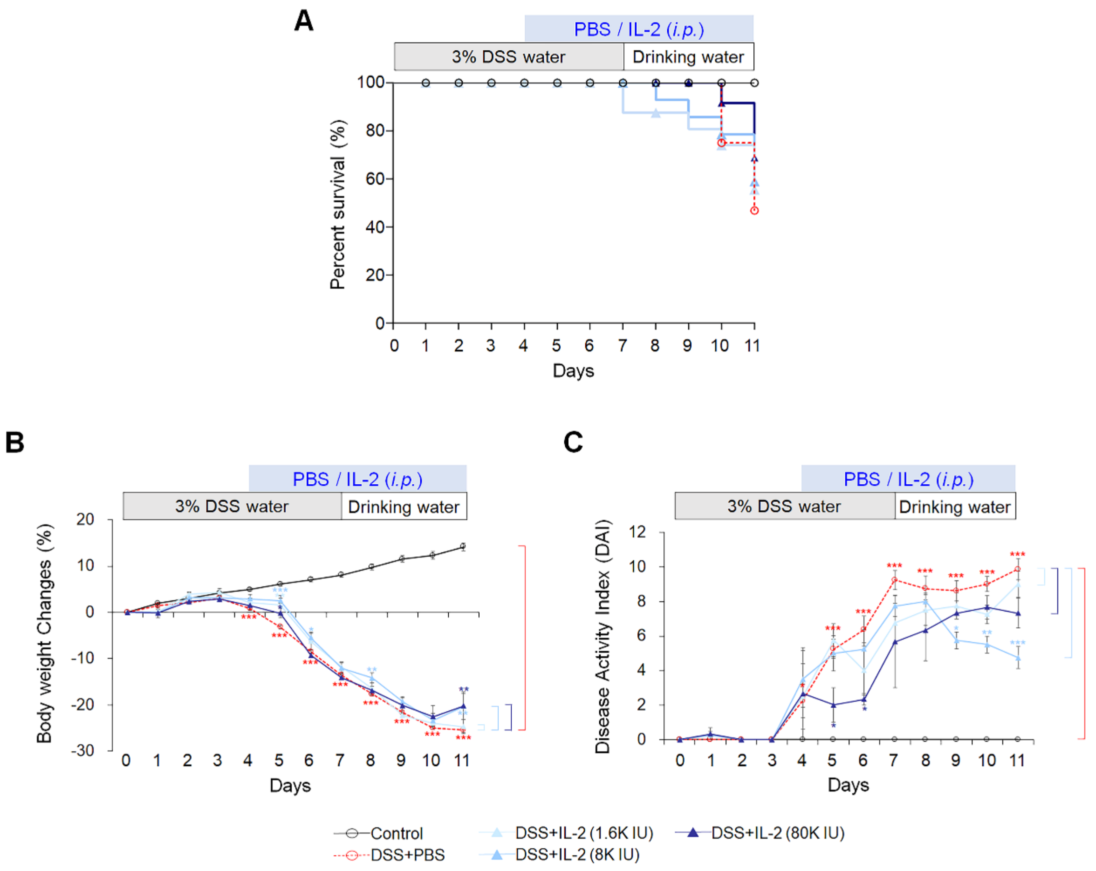

Figure S1. The effect of IL-2 on clinical signs in mice with DSS-induced colitis appears to be concentration-dependent. The groups of mice used in the study were Control (n = 10), DSS+PBS (n = 20), and DSS+IL-2 (1.6K, 8K, and 80K IU, n = 7 for each group). (A) Kaplan-Meier survival analysis. (B) Percentage body weight change. (C) Disease activity index (DAI). Data are presented as mean values of replicates ± SEM. ***$p < 0.001$, **$p < 0.01$, and *$p < 0.05$ using one-way ANOVA with a *post hoc* analysis and t-test.

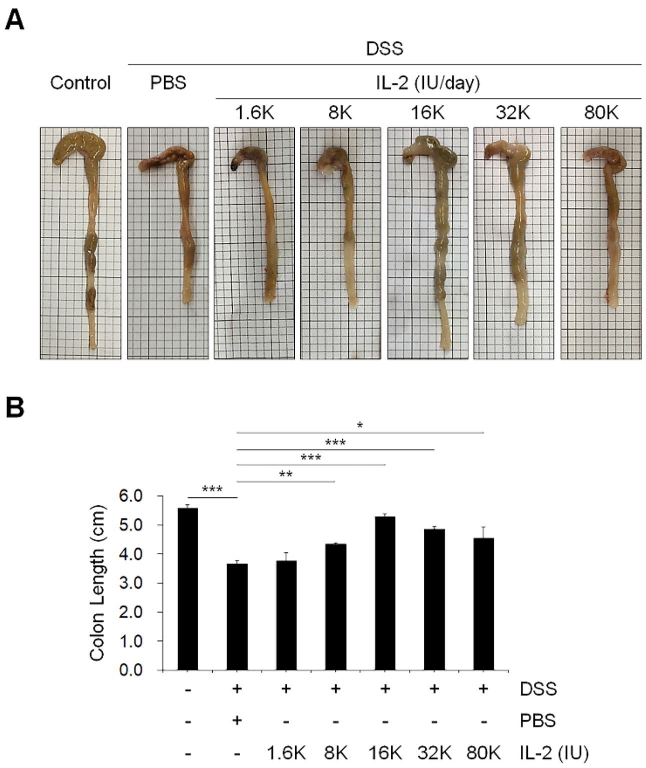

Figure S2. A concentration-dependent effect of IL-2 on colon length in mice with DSS-induced colitis. (A) Representative photographs of the colon. (B) Changes in colon length between control (n = 10), DSS + PBS (n = 10) and IL-2 treated groups (16K and 32K IU, n = 10; 1.6K, 8K and 80K IU, n = 7). Data are presented as mean values of replicates ± SEM. ***$p < 0.001$, **$p < 0.01$, and *$p < 0.05$ according to t-test.

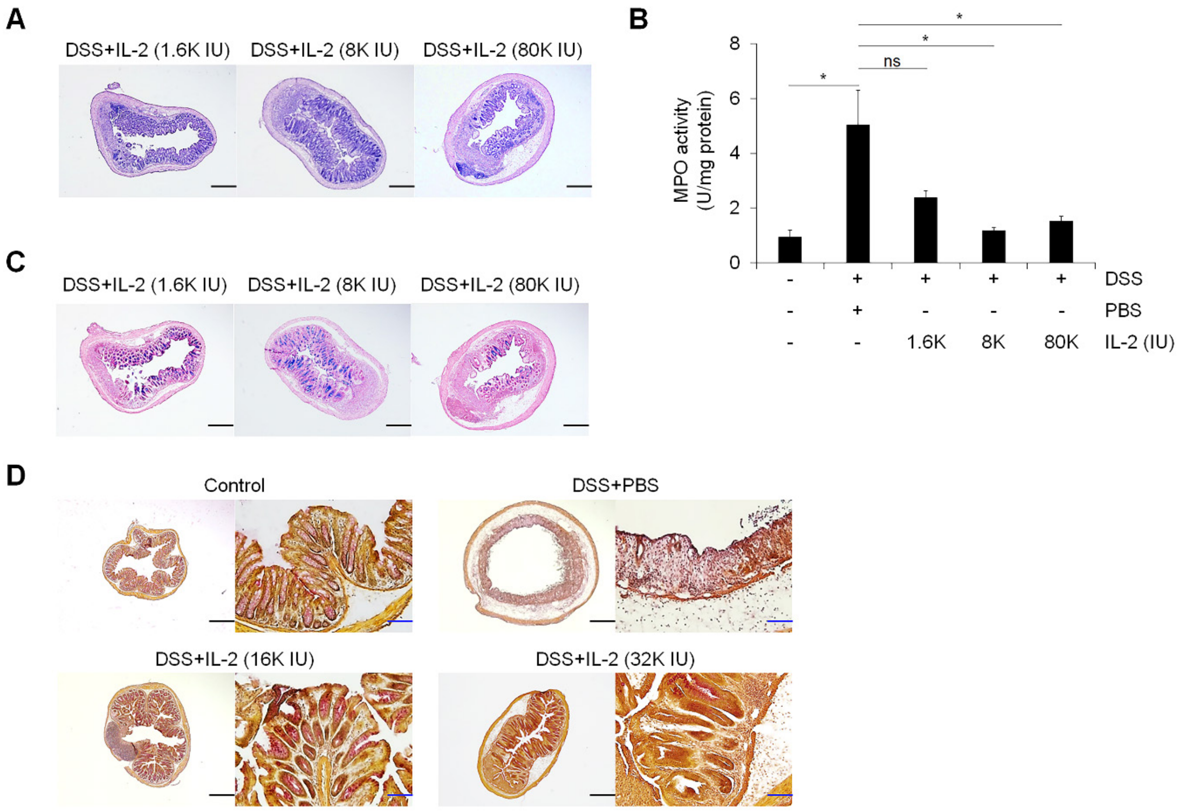

Figure S3. Effect of IL-2 at various concentrations on histopathology of mice with DSS-induced colitis. (A) Histopathological analysis in colon tissue of DSS+IL-2 (1.6K, 8K, and 80K IU) by H&E staining. Scale bar, 500 µm. (B) Myeloperoxidase (MPO) activity in colon tissue of control, DSS+PBS and DSS+IL-2 (1.6K, 8K, and 80K IU, n = 3 for each group) (C) AB-PAS staining to evaluate mucin in DSS+IL-2 (1.6K, 8K, and 80K IU). Scale bar, 500 µm. (D) Mucicarmine staining for the histological visualization of acid mucopolysaccharides in colon tissues of Control, DSS+PBS, and DSS+IL-2 (16K and 32K IU) mice. Scale bar, black 500 µm and blue 100 µm. Data are presented as mean values of replicates ± SEM. ns: not significant, *$p < 0.05$ according to t-test.

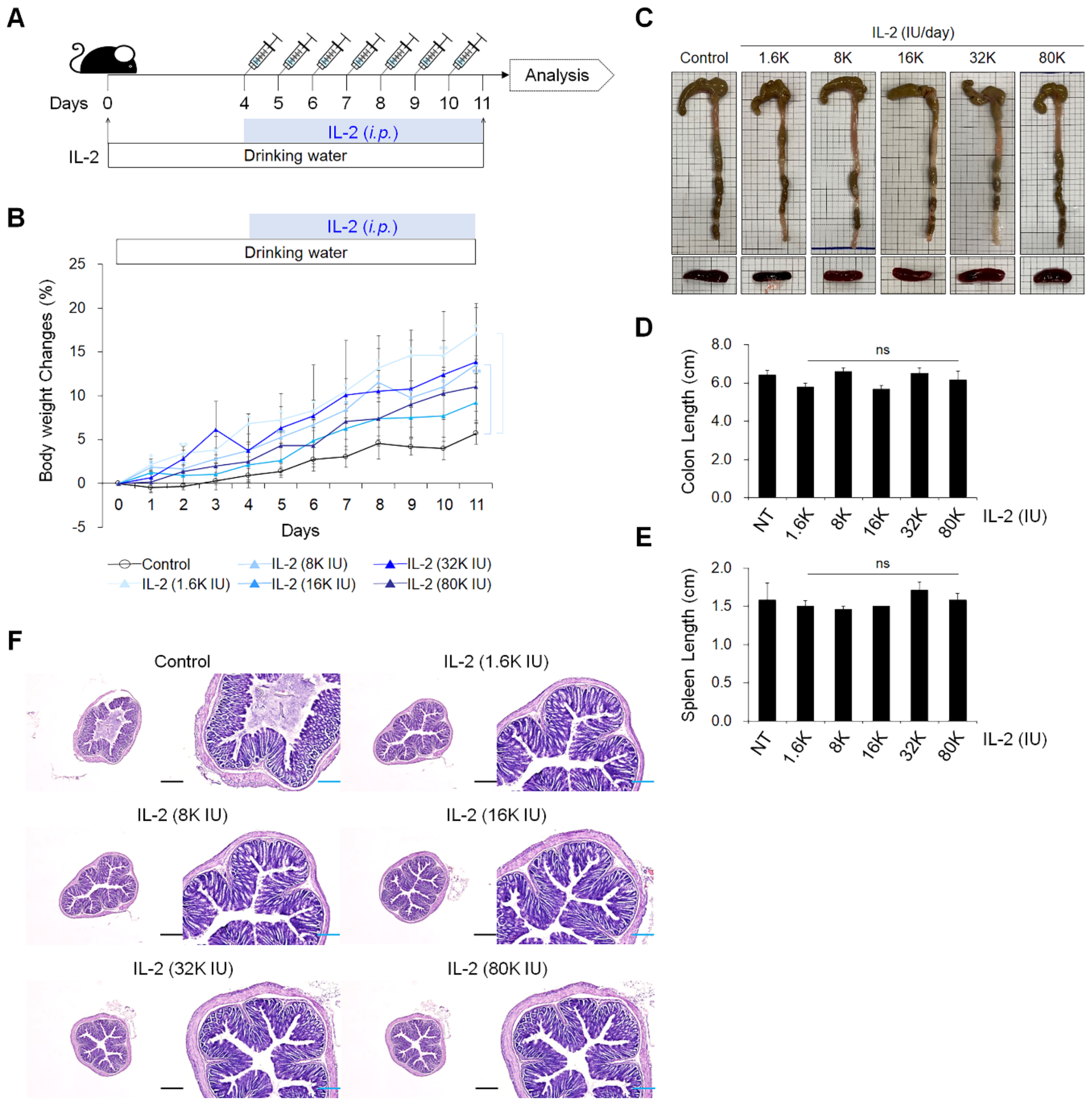

Figure S4. Effect of IL-2 only treatment on the control mice. (A) Schematic diagram of the experimental design. (B) Percentage body weight change (n ≥ 3 for each group). (C) Representative photographs of the colon (upper) and spleen (bottom) (D) Changes in colon length between groups. (E) Changes in spleen length. (F) Histological analysis in colon tissue using H&E staining. Scale bar, black 500 μm and blue 200 μm. Data are presented as mean values of replicates ± SEM. ns: not significant, $^{**}p < 0.01$, and $^{*}p < 0.05$ using one-way ANOVA with a *post hoc* analysis and t-test.

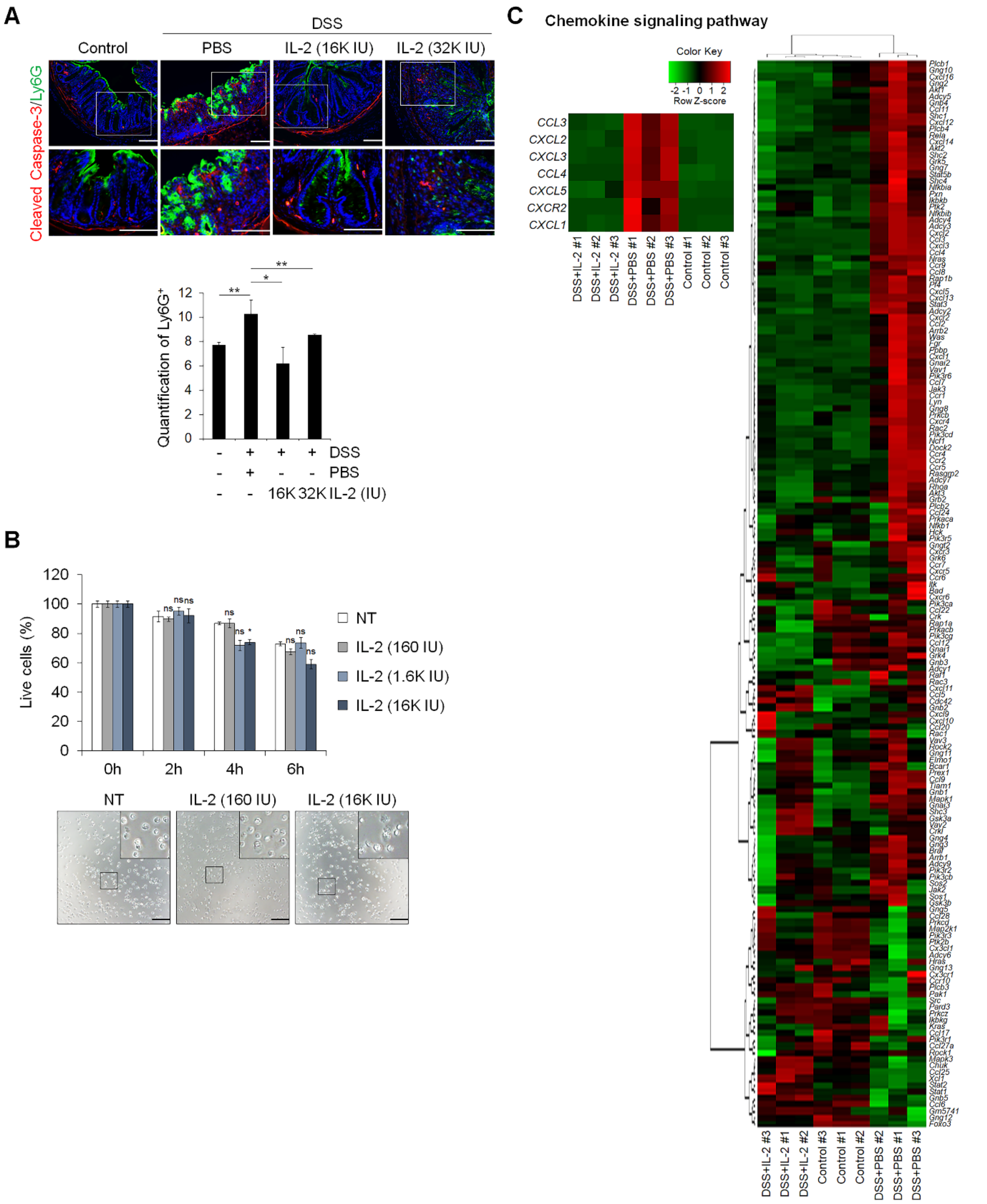

Figure S5. Effect of low-dose IL-2 treatment on neutrophils. (A) Representative immunofluorescence images of infiltrated neutrophils in colon tissue and the integrated density of Ly6G$^+$ cells per area (n = 3 for each group). Scale bar=100 μm. (B) Viability assay of neutrophils after IL-2 treatment *in vitro*. Scale bar=500 μm. (C) Heat-map of chemokine

signaling pathway-related genes (left) and chemokines related to neutrophil infiltrations (right). Data are presented as mean values of replicates ± SEM. ns: not significant, $^{**}p < 0.01$, and $^{*}p < 0.05$ according to t-test.

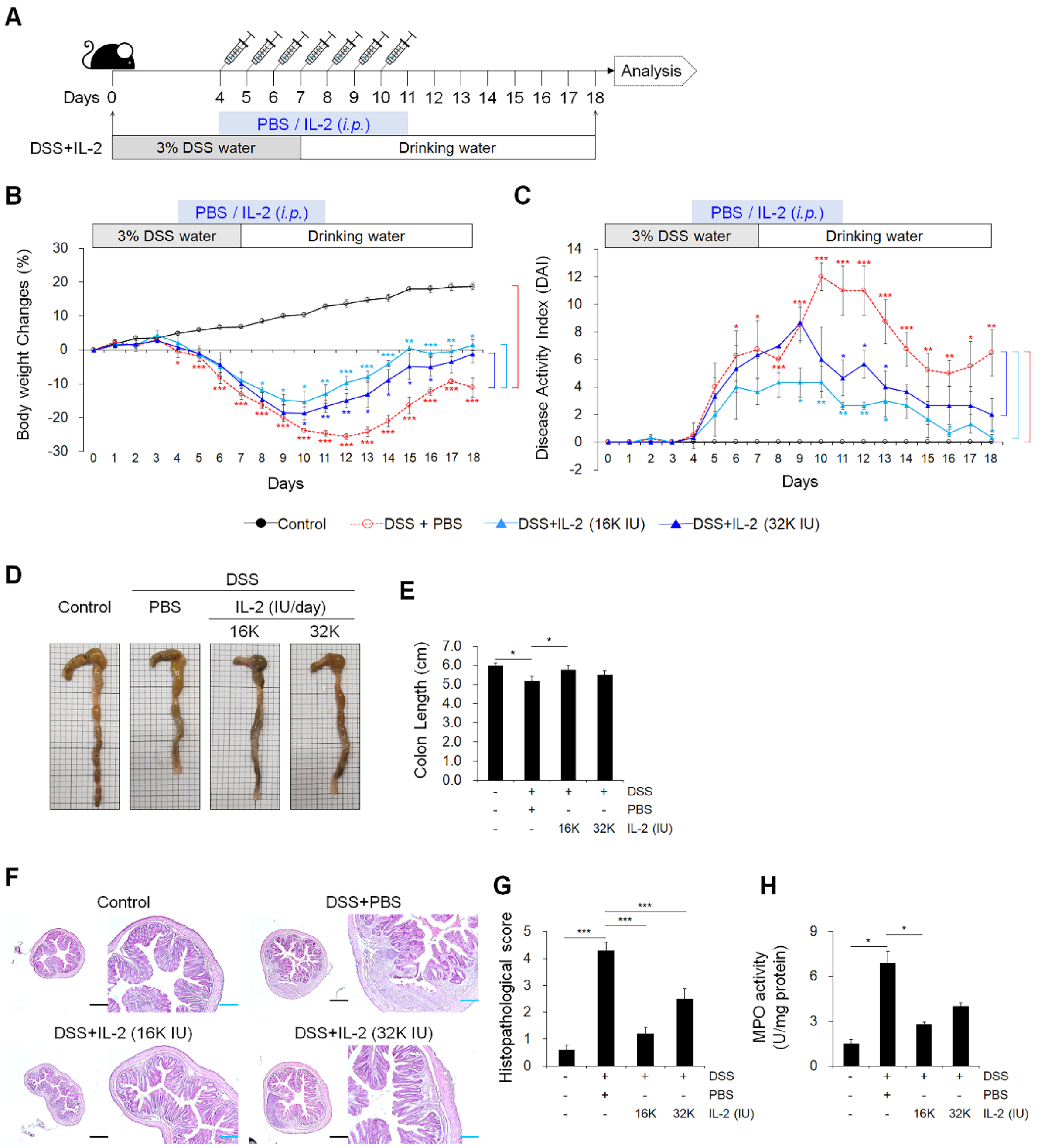

Figure S6. Reverting effect of low-dose IL-2 treatment on the DSS-induced colitis mice. The groups of mice used in the study were Control (n = 5), DSS+PBS (n = 10), DSS+ IL-2 (16K IU and 32K IU, n = 10 for each group). (A) Schematic diagram of the experimental design. (B) Percentage body weight change. (C) Disease activity index (DAI). (D) Representative photographs of the colon. (E) Changes in colon length between groups. (F) Histopathological analysis in colon tissue using H&E staining. Scale bar, black 500 µm and blue 200 µm. (G)

Histopathological changes scored for colon tissue. (H) Myeloperoxidase (MPO) activity of colon tissue. Data are presented as mean values of replicates ± SEM. ***$p < 0.001$, **$p < 0.01$, and *$p < 0.05$ using one-way ANOVA with a *post hoc* analysis and t-test.

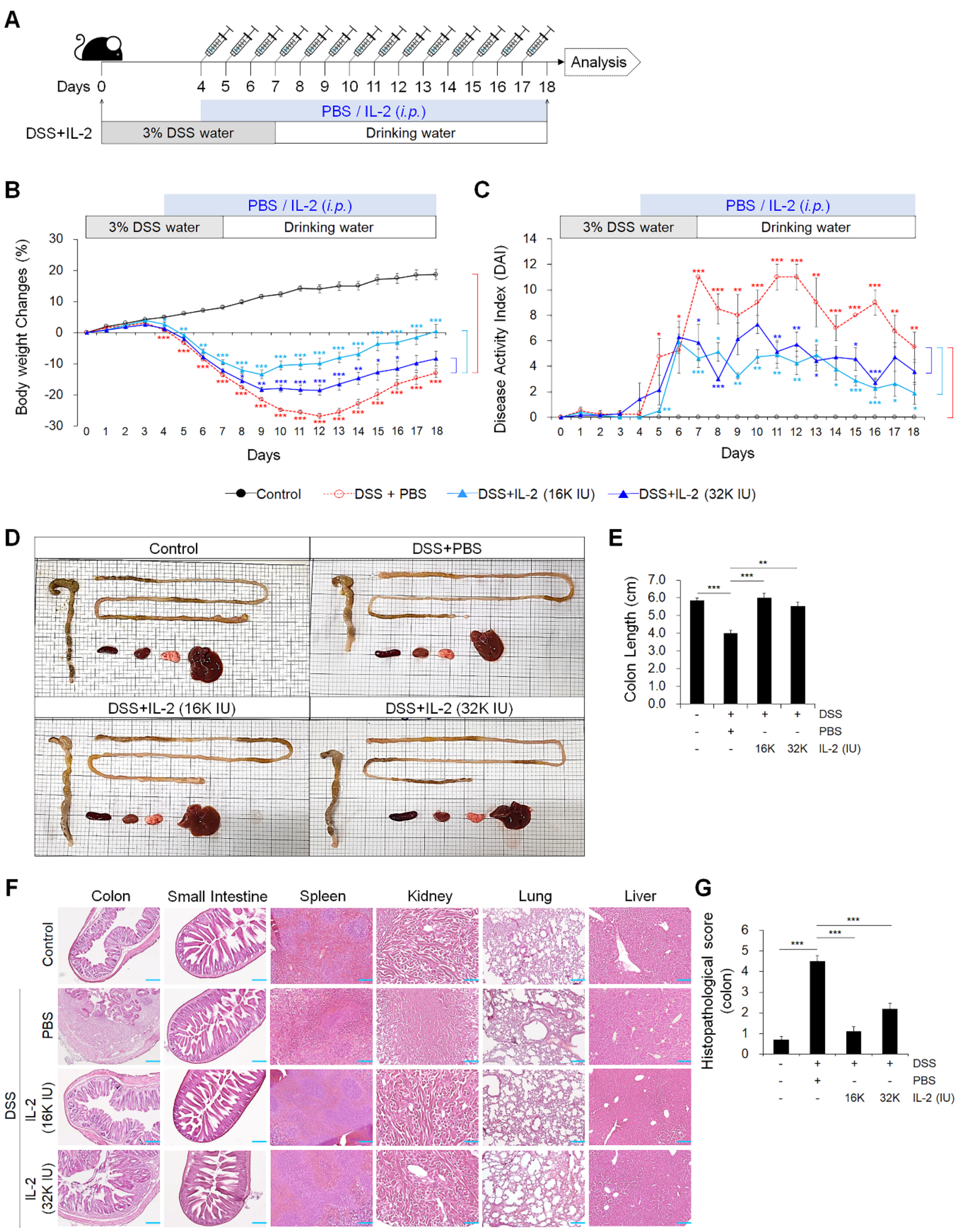

Figure S7. Effect of long-term IL-2 treatment for 2 weeks in mice with DSS-induced colitis. The study groups were Control (n = 5), DSS+PBS (n = 10), DSS+ IL-2 (16K IU and 32K IU, n = 10 for each group). (A) Schematic diagram of the experimental design. (B) Percentage of

body weight change. (C) Disease activity index (DAI). (D) Representative photographs of the colon, small intestine, spleen, kidney, lung and liver. (E) Changes in colon length between groups. (F) Histopathological analysis in tissues using H&E staining. Scale bar, 200 µm. (G) Histopathological changes scored for colon tissue. Data are presented as mean values of replicates ± SEM. ***$p < 0.001$, **$p < 0.01$, and *$p < 0.05$ using one-way ANOVA with a *post hoc* analysis and t-test.

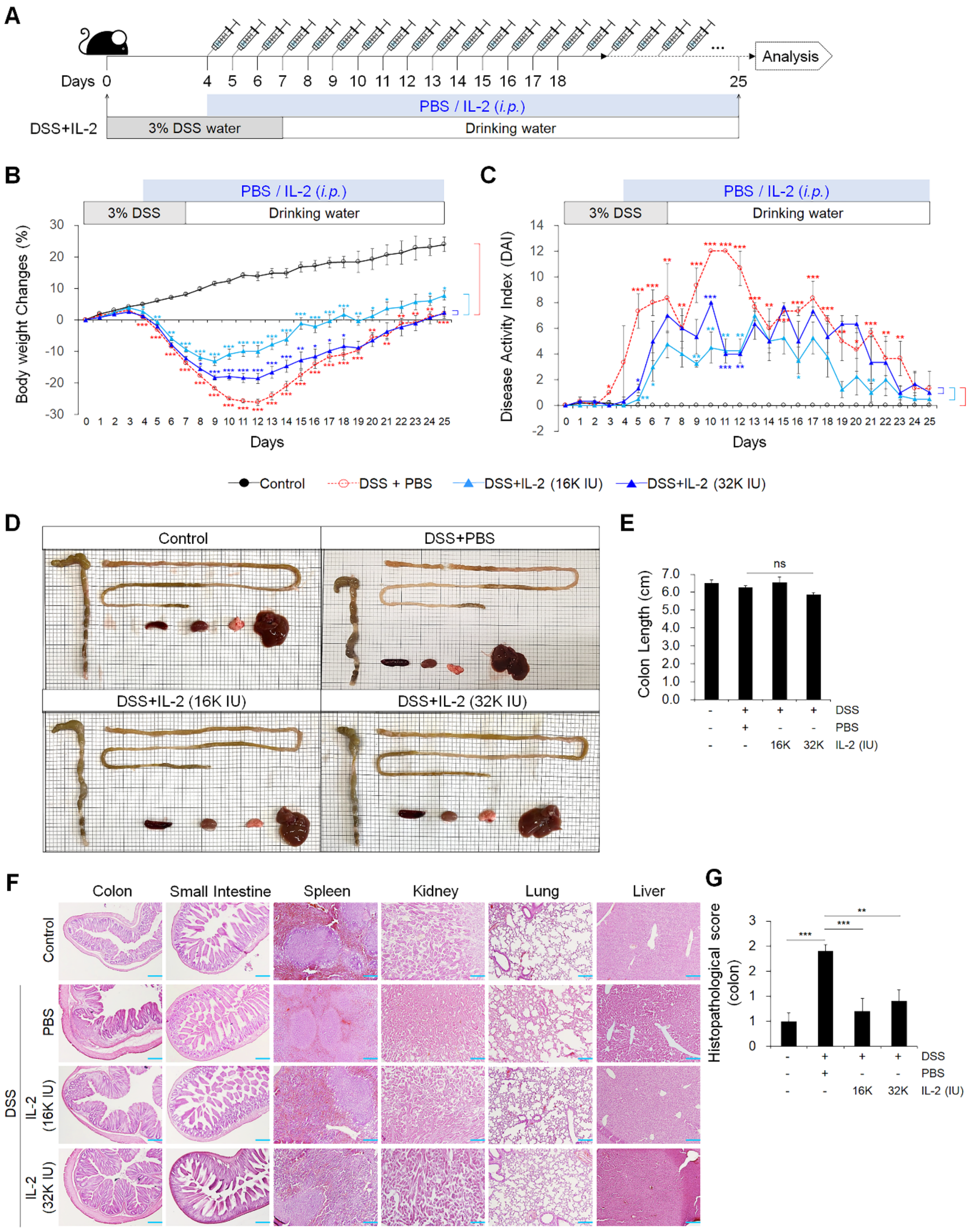

Figure S8. Effect of long-term IL-2 treatment for 3 weeks in mice with DSS-induced colitis. The study groups were Control (n = 5), DSS+PBS (n = 10), DSS+ IL-2 (16K IU and 32K IU, n = 10 for each group). **(A)** Schematic diagram of the experimental design. (B) Percentage

body weight change. (C) Disease activity index (DAI). (D) Representative photographs of the colon, small intestine, spleen, kidney, lung and liver. (E) Changes in colon length between groups. (F) Histopathological analysis in tissues using H&E staining. Scale bar, 200 μm. (G) Histopathological changes scored for colon tissue. Data are presented as mean values of replicates ± SEM. ***$p < 0.001$, **$p < 0.01$, and *$p < 0.05$ using one-way ANOVA with a *post hoc* analysis and t-test.

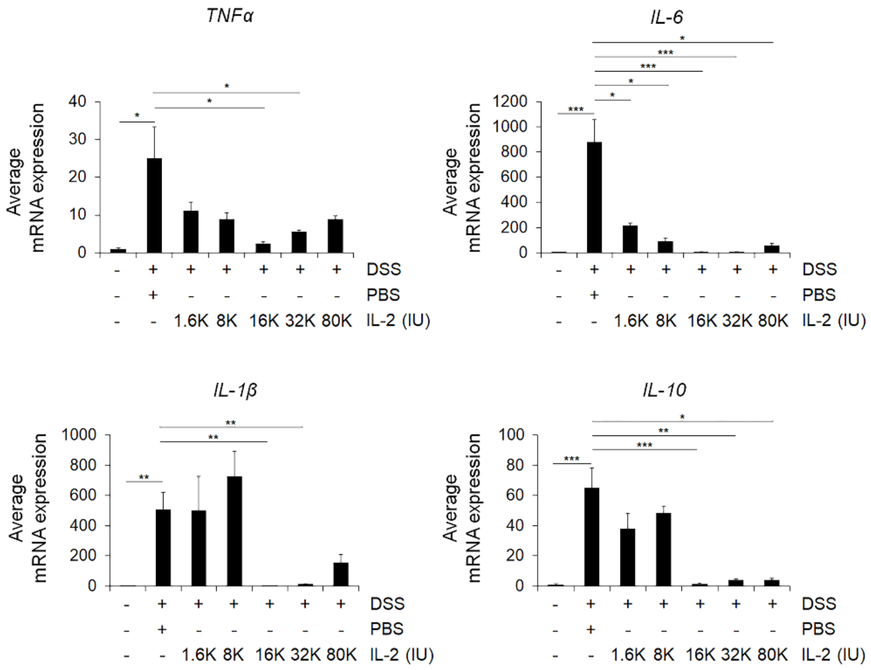

Figure S9. The concentration-dependent effect of IL-2 on pro-inflammatory responses in mice with DSS-induced colitis. The expression levels of the inflammatory cytokines *TNF-α, IL-6, IL-1β, and IL-10* by qPCR analysis in Control (n = 10), DSS+PBS (n = 10), and DSS+IL-2 (16K and 32K IU, n = 10; 1.6K, 8K, and 80K IU, n = 7 for each group). Data are presented as mean values of replicates ± SEM. ***$p < 0.001$, **$p < 0.01$, and *$p < 0.05$ according to t-test.

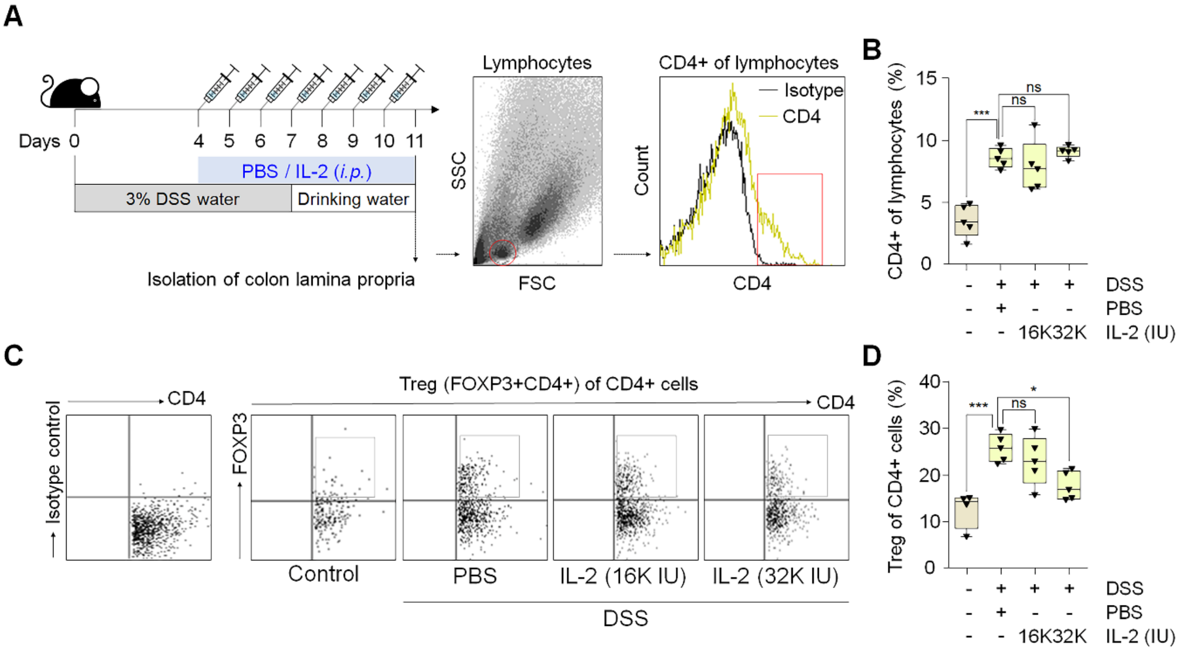

Figure S10. Effect of low-dose IL-2 on the regulatory T cells (Tregs) in colon lamina propria of DSS-induced colitis. (A) Experimental scheme for the isolation of colon lamina propria from DSS-induced colitis and flow cytometry analysis of $CD4^+$ T cells. (B) Percentage of $CD4^+$ T cells in lymphocytes. (C) Representative flow cytometry dot-plots showing Tregs ($FOXP3^+CD4^+$) cells in $CD4^+$ T cells (D) Percentage of Tregs ($FOXP3^+CD4^+$) cells in $CD4^+$ T cells. Percentage data are presented as mean values of replicates ± SEM. ns: not significant, $***p < 0.001$, and $*p < 0.05$ according to t-test.

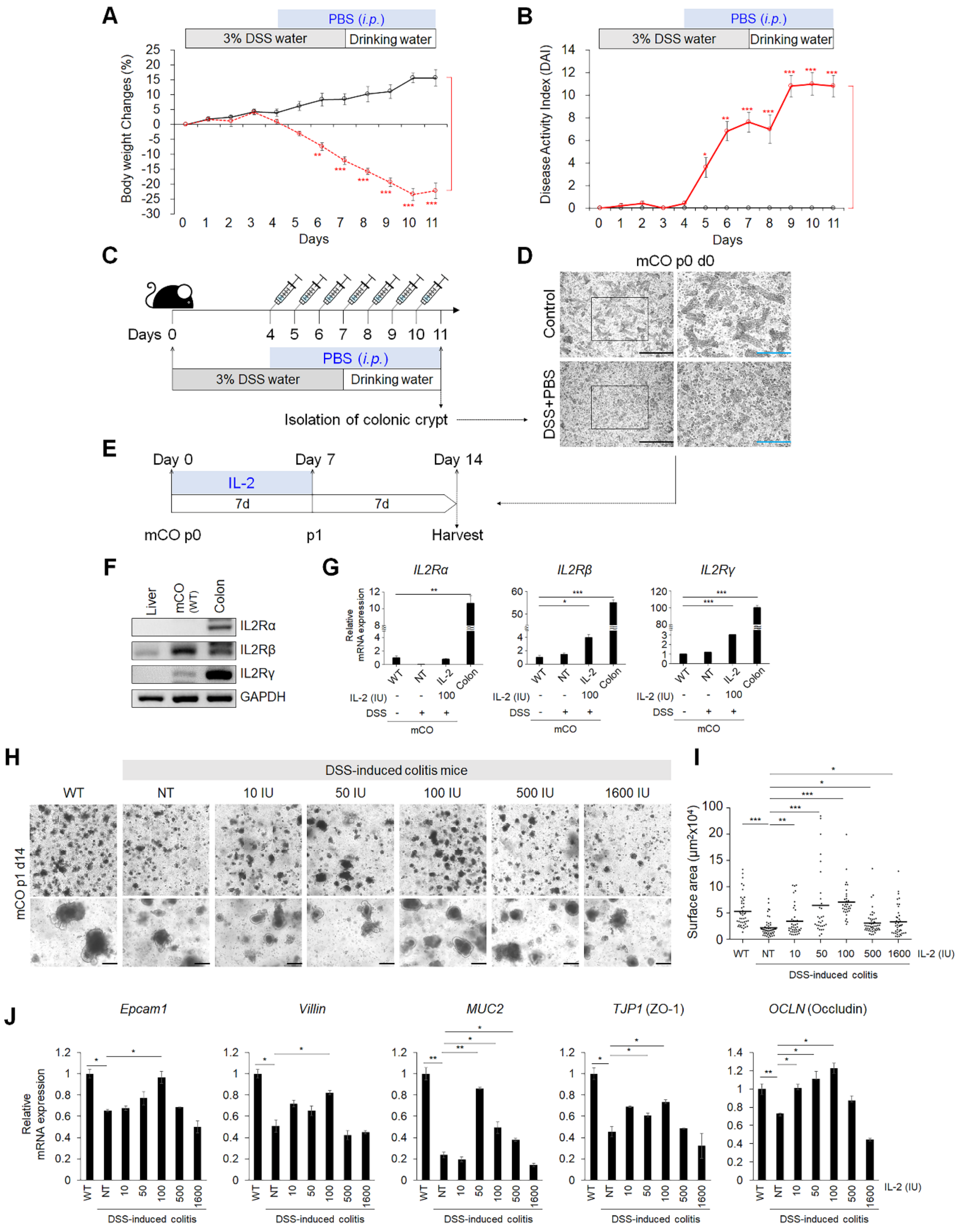

Figure S11. Effect of low-dose IL-2 on mouse colonoids (mCOs) derived from DSS-induced colitis mice. The study groups were Control (n = 3) and DSS+PBS (n = 5) (A) Percentage body weight change. (B) Disease activity index (DAI). (C) Experimental scheme for the

isolation of colonic crypts from DSS-induced colitis. (D) Representative photographs of the colonic crypts from Control and DSS-induced colitis. Scale bar, black 200 μm and blue 100 μm. (E) Schematic diagram of the mCO experimental design. (F) Expression of IL-2R subunits in liver (negative control), mCO and colon analyzed by RT-PCR (G) qPCR analysis of relative gene expression of IL-2R subunits in mCOs. (H) Representative images of the morphology of mCOs with IL-2 treatment (0-1600 IU/ml). Scale bar, black, 200 μm. (I) Quantitative assessment of the surface area of mCOs. ($n \geq 33$ for each group) (J) The relative gene expression level of cell-cell adhesion markers (*Epcam1*), Intestinal epithelial markers (*Villin*), Mucin (*MUC2*) and tight junction markers (*ZO-1* and *occluding*) by qPCR; $n = 5$ per group. Data are presented as mean values of replicates ± SEM. ***$p < 0.001$, **$p < 0.01$, and *$p < 0.05$ according to t-test.

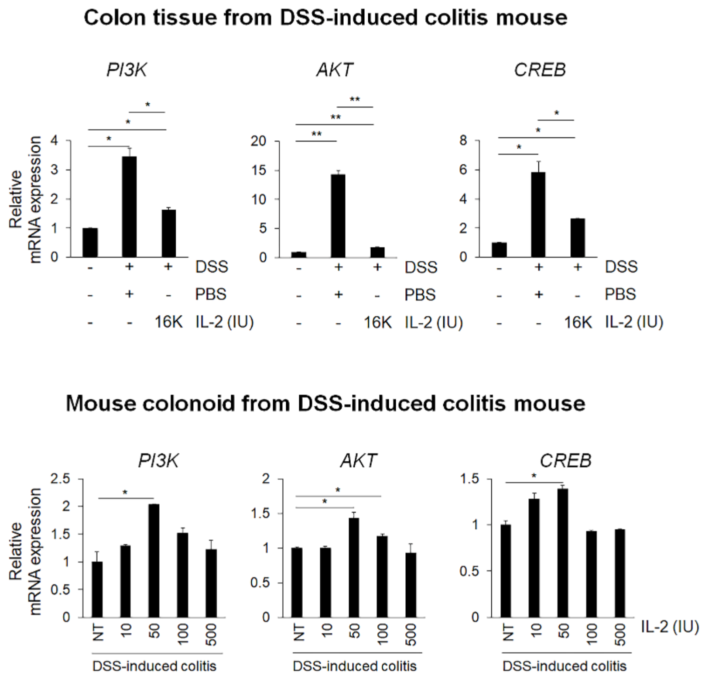

Figure S12. Relative gene expression analysis of PI3K/AKT pathway in colon tissue and mouse colonoids (mCOs) from DSS-induced colitis mice. (A) The expression levels of *PI3K*, *AKT*, and *CREB* using qPCR analysis in colon tissue (Control, DSS+PBS, DSS+IL-2 (16K IU) and mCOs with IL-2 (0, 10, 50, 100, 500 IU/ml); n = 3 per group. Data are presented as mean values of replicates ± SEM. $^{**}p < 0.01$ and $^{*}p < 0.05$ according to t-test.

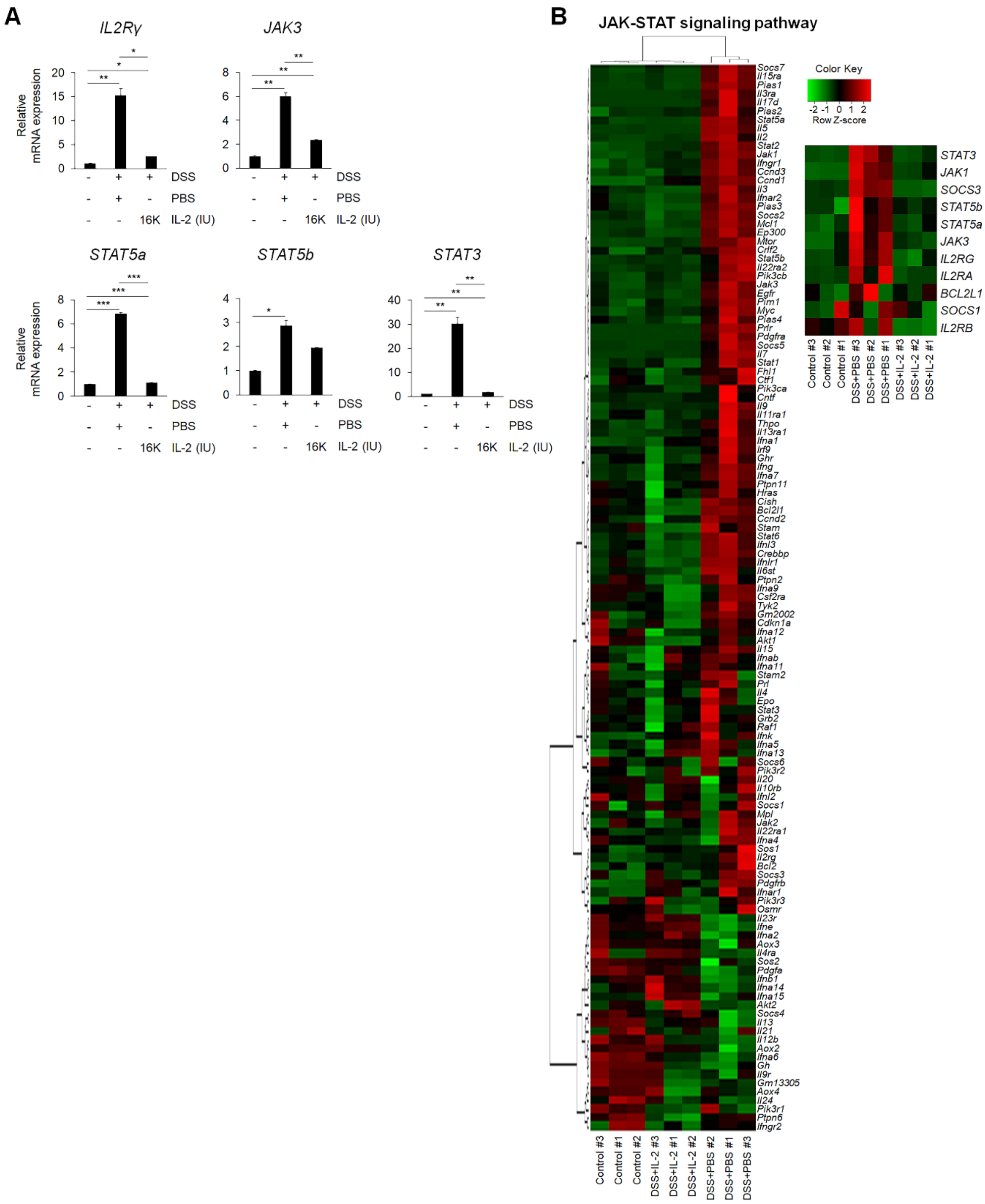

Figure S13. Effect of low-dose IL-2 treatment on the expression of genes involved in the Jak-Stat signaling pathway. (A) The expression levels of the *IL-2Rγ, JAK3, STAT5a, STAT5b, and STAT3* by qPCR analysis in Control, DSS+PBS, and DSS+IL-2 (16K and 32K IU); n = 3 for each group. (B) Heat-map of JAK-STAT signaling pathway-related genes (left) and JAK-

STAT related to IL-2 (right). Data are presented as mean values of replicates ± SEM. ***$p < 0.001$, **$p < 0.01$, and *$p < 0.05$ according to t-test.

Supplementary Table S1. Assessment of disease activity index (DAI)

Body weight loss (%)	Stool	Bleeding	Score
<2%	Normal	No rectal bleeding	0
≥2%-<5%	Softer stool	Weak hemoccult	1
≥5%-<10%	Moderate diarrhoea	Visual blood in stool	2
≥10%-<15%	Diarrhoea	Fresh rectal bleeding	3
≥15%	-	-	4

Supplementary Table S2. Assessment of histopathological scores

Grade	Extent of inflammation	Infiltration neutrophils + lympho-histiocytes	Extent of crypt damage	Crypt abscesses	Sub-mucosal oedema	Loss of goblet cells	Reactive epithelial hyperplasia
0	None	None	None	None	None	None	None
1	Mucosa	Focal	Basal one third	Focal	Focal	Focal	Focal
2	Mucosa+submucosa	Multifocal	Basal two third	Multi-focal	Multi-focal	Multi-focal	Multifocal
3	Mucosa+submucosa +muscle layer	Diffuse	Entire crypt damage		Diffuse	Diffuse	Diffuse
4	Transmura	-	Crypt damage+ ulceration				

Supplementary Table S3. List of the primers used in this study.

Gene	Primer (Forward)	Primer (Reverse)
β-actin	AGC CAT GTA CGT AGC CAT CC	CTC TCA GCT GTG GTG GTG AA
Gapdh	GTG TTC CTA CCC CCA ATG TG	TGT CAT TGA GAG CAA TGC CAG
Tnfα	GAA CTG GCA GAA GAG GCA CT	AGG GTC TGG GCC ATA GAA CT
Il-6	AGT TGC CTT CTT GGG ACT GA	CAG AAT TGC CAT TGC ACA AC
Il-1β	GCC CAT CCT CTG TGA CTC AT	AGG CCA CAG GTA TTT TGT CG
Il-17a	GCT CCA GAA GGC CCT CAG A	AGC TTT CCC TCC GCA TTG A
Ifnγ	GGC CAT CAG CAA CAA CAT AAG CGT	ACC TGT GGG TTG TTG ACC T
iNos	CGA GGA GCA GGT GGA AGA CT	TGG AAC TCT GGG CTG TCA GA
Cox-2	AAC CGA GTC GTT CTG CCA AT	CCT GGG ATG GCA TCA GTT TT
Muc2	TCG GTC TCC AAC ATC ACC TG	AGC AGA GCA AGG GAC TCT GG
Tjp1 (Zo-1)	CAC AAG GAG CCA TTC CTG AAG	ATC ACT AGG GGG CTC AGC AG
Occludin	AGG ACG GAC CCT GAC CAC TA	CCT GCA GAC CTG CAT CAA AA
Epcam1	TCA TCG CTG TCA TTG TGG TG	GTC CGA GCT CTT CTG CCA CT
Villin	AGG TTA TGA GCC CGA AAG TG	ATG TTT TGT TGC TTC CAT CG
Pi3k	GGG GAA TGA AAA TAC CGA AGA	TAC GGA GCA GGC ATA GCA G
Pik3cd	AGT TTG GAA TCA ACC GAG AGC	CGA AGA GAT GGA GGA AAA GCA
Pik3ap1	AGT GTG AGC AGT GGA ATG GAG	CGG AGG AGG ATG GAA GGA CT
Pik3r5	AGG CAG AAC CCT AAA TCC AAA	GCT CTT CTC TGG CTT GTA GCA
Akt	TGT GGC AGG ATG TGT ATG AGA	GTA GGA GAA CTG GGG GAA GTG
Akt3	CCG AAC ACT CTC TTC AGA TGC	ATC GGG TGT CTG TTT CAG ATG
Creb	CAC AGA CCA CTG ATG GAC AGC	CTT CTT TCT ACG ACA TTC TCT TGC
Creb3l2	TCC CCA TTT CCT TAT CTC CAA	AGT GTT TCG TTC CCT TCC AGT
Creb5	GGA AGG TCT GGG TGA TGT C	TGT TGG ATA ACT TGC TGC TGA
NFkBp65	ATG AAC TTG TGG GGA AGG ACT	GGG GTT ATT GTT GGT CTG GAT
Il-2rα	ACT TCC CAC AAC CCA CAG AA	TAG GTG AAT GCT TGG CGT CT
Il-2rβ	AAG CCT CAA GCA GAG ACA GC	GCC AGA AAA ACA ACC AAG GA
Il-2rγ	TGC CTA GTG TGG ATG AGC TG	GTG CCA ACA GGG ATA AGC AC
Jak3	GTG GAA GAC CCG GAT AGC AG	GTG CAG CCA GTA AGA CAG CA
Stat5a	ACC GAA ACC TCT GGA ATC TG	TCA GGG ACC ACT TGC TTG AT
Stat5b	GTG AAG CCA CAG ATC AAG CA	TCG GTA TCA AGG ACG GAG TC
Stat3	CCC CGT ACC TGA AGA CCA AG	CCG AGG TCA GAT CCA TGT CA

Supplementary Table S4. List of antibodies used in this study

Antibodies	Catalog No.	Company	Dilution
immunohistochemistry			
anti-TNFα	ab6671	abcam	1:200
anti-IL-17	ab79056	abcam	1:200
anti-IFNγ	ab9657	abcam	1:100
anti-CD11b	ab133357	abcam	1:200
anti-F4/80	ab6640	abcam	1:50
anti-Mucin 2	sc-7314	Santa Cruz Biotechnology	1:50
anti-Cleaved Caspase-3	9664	CST	1:500
immunofluorescence			
anti-ZO-1	61-7300	Thermo Fisher Scientific	1:200
anti-Claudin-1	ab15098	abcam	1:200
anti-E-cadherin	AF648	R&D	1:200
anti-phospho-AKT (S473)	9271	CST	1:50
anti-Ly6G	ab25377	abcam	1:100
anti-iNOS	ab49999	abcam	1:200
Western blot			
anti-PI3 Kinase p85	4257	CST	1:1000
anti-AKT	2920	CST	1:1000
anti-phospho-AKT (S473)	9271	CST	1:1000
anti-NF-κB p65	8242	CST	1:1000
anti-phospho-NF-κB p65 (Ser536)	3033	CST	1:1000
anti-β-actin	A5441	Sigma	1:2000
FACS			
PE-CF594 Rat Anti-Mouse Foxp3	562466	BD Biosciences	1:100
FITC Rat Anti-Mouse CD4	561831	BD Biosciences	1:100
